# Neural differentiation of glioblastoma cell lines via a herpes simplex virus thymidine kinase/ganciclovir system driven by a glial fibrillary acidic protein promoter

**DOI:** 10.1371/journal.pone.0253008

**Published:** 2021-08-09

**Authors:** Elizabeth Wei-Chia Luo, Meng-Lin Liao, Chung-Liang Chien

**Affiliations:** 1 Graduate Institute of Anatomy and Cell Biology, College of Medicine, National Taiwan University, Taipei, Taiwan; 2 Department of Bioengineering, University of California, Los Angeles, California, United States of America; 3 School of Medicine, College of Medicine, I‐Shou University, Kaohsiung, Taiwan; Medical University of Lodz, POLAND

## Abstract

Glioblastoma is a malignant brain tumor with poor prognosis that rapidly acquires resistance to available clinical treatments. The herpes simplex virus thymidine kinase/ganciclovir (HSVtk/GCV) system produces the selective elimination of HSVtk-positive cells and is a candidate for preclinical testing against glioblastoma via its ability to regulate proliferation and differentiation. Therefore, in this study, we aimed to establish a plasmid encoding the HSVtk/GCV system driven by a glial fibrillary acidic protein (GFAP) promoter and verify its possibility of neural differentiation of glioblastoma cell line under the GCV challenge. Four stable clones—N2A-pCMV-HSVtk, N2A-pGFAP-HSVtk, U251-pCMV-HSVtk, and U251-pGFAP-HSVtk—were established from neuronal N2A and glioblastoma U251 cell lines. *In vitro* GCV sensitivity was assessed by MTT assay for monitoring time- and dosage-dependent cytotoxicity. The capability for neural differentiation in stable glioblastoma clones during GCV treatment was assessed by performing immunocytochemistry for nestin, GFAP, and βIII-tubulin. Under GFAP promoter control, the U251 stable clone exhibited GCV sensitivity, while the neuronal N2A clones were nonreactive. During GCV treatment, cells underwent apoptosis on day 3 and dying cells were identified after day 5. Nestin was increasingly expressed in surviving cells, indicating that the population of neural stem-like cells was enriched. Lower levels of GFAP expression were detected in surviving cells. Furthermore, βIII-tubulin-positive neuron-like cells were identified after GCV treatment. This study established pGFAP-HSVtk-P2A-EGFP plasmids that successfully ablated GFAP-positive glioblastoma cells, but left neuronal N2A cells intact. These data suggest that the neural differentiation of glioblastoma cells can be promoted by treatment with the HSVtk/GCV system.

## Introduction

Glioblastoma is a devastating, uniformly lethal primary brain tumor. The standard treatment for glioblastoma is surgery followed by radiotherapy and chemotherapy, but tumor recurrence is observed in most patients [1]. In fact, the median survival time for patients is only 12–15 months, despite undergoing the maximum treatment [2]. A contributor to the tumor reoccurrence is a cell population with cancer stem cell properties present in glioblastoma.

Previous studies revealed that malignant tumors are initiated by a population of tumor cells sharing similar biological properties with normal adult stem cells [3, 4]. These tumor cells, known as cancer stem cells, have self-renewal phenotypes, which may be the cause of resistance to the current standard care of concomitant chemoradiotherapy [5]. Treatment with anticancer drugs and irradiation can cause cancer cells to die by apoptosis, but cancer stem cells can survive and are related to cancer recurrence [6, 7]. Therefore, controlling the proliferation and differentiation of cancer stem-like cells is a key factor in controlling malignant cancer [4, 8, 9]. Inducing differentiation and apoptosis of cancer stem cells offers a promising strategy for the management and eradication of different types of cancers [10]. Similarly, regulating the differentiation of glioma cells has been suggested as a strategy for controlling glioblastoma [11]. Recent research revealed that glioma-initiating cells (GIC) isolated from human glioblastoma differentiated and increased their radiosensitivity when treated with rapamycin [12]. In addition, autophagy was suggested to play an essential role in the regulation of self-renewal, differentiation, and tumorigenicity of GIC [13].

Ganciclovir (GCV) is an antiviral medication used to treat infections from the herpes family. GCV is first phosphorylated to GCV monophosphate by a viral kinase encoded by the cytomegalovirus (CMV) gene. Afterwards, GCV is metabolized to the triphosphate form by guanylate kinase and phosphoglycerate kinase. GCV-5′-triphosphate inhibits the replication of viral DNA. This inhibition includes a selective inhibition of the viral DNA polymerase and leads to disruption of DNA synthesis and inhibition of cell proliferation [14–16]. Since the herpes simplex virus thymidine kinase (HSVtk)/GCV system produces selective elimination of HSVtk-positive cells, it has been used in a clinical gene therapy trial as a therapeutic gene to kill cancer cells. Several tumor cell lines, including sarcoma, melanoma, and colon carcinoma, displayed sensitivity to this suicide gene approach [17]. This gene therapy has also shown potential as a treatment for cervical cancer [18] and gastrointestinal cancer [19]. Besides, neural stem/progenitor cells derived from human induced pluripotent stem cells (hiPSCs) with the HSV-TK/GCV suicide gene system showed prolonged survival time and the bystander killing in an orthotopic xenograft mouse model of glioblastoma upon GCV treatment. However, stable constitutive gene expression of HSV-TK was highly cytotoxic and difficult to sustain in hiPSCs and the recurrence occurred 2–3 weeks after GCV treatment [20].

As mentioned, HSVtk-positive cells can be selectively ablated by the HSVtk/GCV system. However, controlling HSVtk expression in mammalian cells is also essential to avoid any influence on other nontarget cell populations. For this purpose, the delivery of the HSVtk/GCV system using embryonic stem cells is a possible approach. Embryonic stem cells have the potential for use in cell replacement therapies, but the transplantation of differentiated cells harbors the risk of teratoma formation. This problem was overcome by establishing a negative selection system that permitted selective removal of undifferentiated stem cells [21]. The HSVtk gene under the control of the Oct4 promoter allowed the destruction of undifferentiated embryonic stem cells by GCV treatment and provided a pure population of completely differentiated cells [22]. The HSVtk/GCV system has been used to ablate undifferentiated cells, but the possibility that it could be used to ablate an unwanted differentiated lineage is still not clear.

Glial fibrillary acidic protein (GFAP), a type III intermediate filament protein, is expressed mainly in astroglia [23, 24], and its expression is increased upon maturation of astrocytes [25]. The GFAP promoter is a 2.2-kb promoter region of the GFAP gene that drives astrocyte-specific expression and regulates the development of astrocytes and CNS physiology [26, 27]. Previous study showed that the HSV-TK gene driven by human GFAP promoter could selectively kill glioma cell lines [28], but did not investigated the possible ability of neural differentiation under GCV treatment. Therefore, the aims of this study were to establish a recombinant plasmid vector bearing the HSVtk/GCV suicide system driven by a GFAP promoter to ablate GFAP-positive tumor cell lines selectively, and to characterize the possible neural differentiation of glioblastoma under GCV challenge.

## Materials and methods

### Plasmid construction

P2A, a 66-bp self-cleaving peptide, was synthesized by oligos annealing with restriction enzyme cutting sites *EcoRI* at the 5′-end and *SalI* at the 3′-end. The forward oligo was 5′-GAATTCGGAAGCGGAGCTACTAACTTCAGCCTGCTGAAGCAGGCTGGAGACGTGGAGGAGAACCCTGGACCT-3′ and the reverse oligo was 5′-GTCGACAGGTCCAGGGTTCTCCTCCACGTCTCCAGCCTGCTTCAGCAGGCTGAAGTTAGTAGCTCCGCTTCC-3′. Next, 100 μM of forward and reverse primers were dissolved in annealing buffer (100 mM KOAc, 30 mM HEPES-KOH, 2 mM MgOAc) and annealed in a thermo-cycler (95°C for 4 minutes, 70°C for 10 minutes, 37°C for 20 minutes, and 10°C for 5 minutes at the end). The P2A oligo was then subcloned into pEGFP-N3 (Clontech Laboratories, Mountain View, CA, USA) at restriction enzyme sites: *EcoRI* at the 5′-end and *SalI* at the 3′-end. After pEGFP-N3-P2A was constructed, HSVtk was inserted upstream of P2A. The clone with a full-length 1131-bp HSVtk was purchased from Addgene (Cat. #pTGB008, Addgene, Watertown, MA, USA). The primers used to clamp HSVtk were 5′-CTGCTCGCCGGGATCCATGGCTTCGTACCCCTGC-3′ and 5′-CCATGGTGGCGTCGACAGGTCCAGGGTTCTCCTC-3′. An In-Fusion® HD cloning kit (Clontech Laboratories, Cat. #011614) was used to fuse HSVtk and linearized pEGFP-N3-P2A with *Xhol* at the 5′-end and *EcoRI* at the 3′-end. The sequences of P2A and HSVtk were checked by DNA sequencing provided by Genomics Inc. (New Taipei City, Taiwan) and confirmed in frame. The plasmid pCMV-HSVtk-P2A-EGFP was constructed and confirmed by DNA sequencing.

After pCMV-HSVtk-P2A-EGFP was constructed, the CMV promoter was replaced by the GFAP promoter to construct pGFAP-HSVtk-P2A-EGFP. Plasmid pAAV-GFAP-hChR2(H134R)-mCherry with a full-length 2207-bp GFAP promoter was purchased from Addgene (Cat. #27055, RRID: Addgene_27055). The GFAP promoter region was clamped by a forward primer 5′-GGACTCAGATCTCGAGCCCACCTCCCTCTCTGTG-3′ and a reverse primer 5′-GATCCCGGGCCCGCGGAGGTCCAGGGTTCTCCTC-3′. An In-Fusion HD® cloning kit was used to fuse the GFAP promoter and linearized pCMV-HSVtk-P2A-EGFP together. The sequence of the GFAP promoter was confirmed by DNA sequencing provided by Genomics, Inc.

### Cell culture

The NIH/3T3 cell line (American Type Culture Collection [ATCC], CRL-1658™, Manassas, VA, USA, RRID: CVCL_0594), a mouse fibroblast cell line, was cultured in Dulbecco modified Eagle medium (DMEM; Gibco®, Cat. #11962–092, Waltham, MA, USA) supplemented with 10% fetal bovine serum (FBS; qualified, Gibco®, Cat. #6140–079,) and 1% antibiotic-antimycotic solution (Anti-anti; Gibco®, Cat. #15240–062). The Neuro-2A (N2A) cell line (ATCC, CCL-131™, RRID: CVCL_0470), derived from a spontaneous neuroblastoma tumor of a strain A albino mouse, and the U251 cell line (Sigma-Aldrich, Cat. #09063001, St. Louis, MO, USA, RRID: CVCL_0021), derived from a malignant glioblastoma tumor, were cultured separately in Minimum Essential Medium α (MEM α, Gibco®, Cat. #12571–063) supplemented with 10% heat-inactivated FBS (Gibco®, Cat. #6140–079), 1% of nonessential amino acid (Gibco®, Cat. #11140–050), and 1% Anti-anti (Gibco®, Cat. #15240–062). All cell lines were seeded onto cell culture dishes (Corning, New York, NY, USA) and maintained in a 37°C humidified incubator with 5% CO_2_. Medium was refreshed every 2~3 days.

### Plasmid transfection and promoter expression level assay followed by stable cell clone selection

NIH/3T3 and U251 cells (1 × 10^5^) were seeded on 12-well cell culture plates 1 day before plasmid transfection. In this experiment, Lipofectamine® LTX with PLUS™ reagents (Invitrogen™, Cat. #15338, Waltham, MA, USA) were used. Both NIH/3T3 and U251 cell lines were transfected with 2 μg of pCMV-HSVtk-P2A-EGFP or pGFAP-HSVtk-P2A-EGFP plasmid. Since the transfection efficiency of the two plasmids into N2A cells was low using Lipofectamine, N2A cells were transfected using the Neon® electroporation transfection system (Thermo Fisher Scientific, Cat. #MPK5000, Waltham, MA, USA) and cell density was increased to 5 × 10^6^ cells/ml. Before transfection, N2A cells were trypsinized to individual cells dissolved in buffer R, and 2 μg of pCMV-HSVtk-P2A-EGFP or pGFAP-HSVtk-P2A-EGFP plasmid DNA were added. Electroporation parameters for N2A cells were 1200 V, 50 ms once. Cells were reseeded into 12-well cell culture plates containing 1 ml electroporation transfection medium per well.

To analyze the promoter expression level of pCMV-HSVtk-P2A-EGFP and pGFAP-HSVtk-P2A-EGFP between glial cell lines and neuron/fibroblast cell lines, the images of transfected cells were acquired under an inverted fluorescence microscope (DM IRM HC; Leica, Wetzlar, Germany). Cells that emitted strong green fluorescence were identified as successfully transfected with promoter expression. The promoter expression level was calculated as followed:

Promoter expression level = (*the number of green fluorescent cells / total cell number under phase image*).

After transfection, 400 μg/ml G418 sulfate (Millipore, Cat. # 345810, Darmstadt, Germany) was added to the medium to select successfully transfected U251 cells. G418 sulfate selection lasted for 4 weeks and the cell population was also enriched by fluorescence-activated cell sorting (FACS; BD Bioscience, BD FACSAria™ IIIu, San Jose, CA, USA). Thereby, the U251-pCMV-HSVtk and U251-pGFAP-HSVtk stable clones were established. For N2A stable clone selection, 500 μg/ml G418 sulfate were applied to transfected N2A cells. After 4 weeks of G418 selection, surviving cells with green fluorescence of the N2A-pCMV-HSVtk cells were enriched by FACS and surviving cells of N2A-pGFAP-HSVtk cells were selected. Thus, the N2A-pCMV-HSVtk and N2A-pGFAP-HSVtk stable clones were established for use in further studies. After stable clones were established, G418 was still present for the maintenance of all stable clone cells, but not in the experimental cells.

### Western blot analysis

For protein extraction, cells were trypsinized and homogenized with radioimmunoprecipitation assay buffer (RIPA buffer; Cell Signaling Technology, Cat. #9806, Danvers, MA, USA), 1 × phosphatase inhibitor (Roche Applied Science, Cat. #4906837001, Mannheim, Germany), and 1 × protease inhibitor (Roche Applied Science, Cat. # 4693116001). Cell lysates were centrifuged at 14,000 rpm for 30 minutes at 4°C, and supernatant was collected. Protein samples with 1 × protein sample buffer were boiled at 97°C for 10 minutes and then placed onto ice immediately. The prepared protein samples were stored at –20°C for further analysis. Protein samples (40 μg/lane) were resolved in 10% sodium dodecyl sulfate polyacrylamide gel electrophoresis (SDS-PAGE), separated at 120 V for 2 hours, and transferred to polyvinylidene fluoride (PVDF) membranes (Millipore, Molsheim, France) at 100 V for 90 minutes. Subsequently, the PVDF membrane with transferred proteins was blocked with a blocking buffer (5% skim milk dissolved in 1 × Tris-buffered saline with Tween-20 [TBST]; 0.1% Tween-20 in 1 × TBS) for 1 hour at room temperature. After blocking, the PVDF membrane was incubated with primary antibodies (see S1 Table) diluted in a blocking buffer overnight at 4°C. After washing, the PVDF membrane was incubated with horseradish peroxidase (HRP)-conjugated secondary antibodies diluted in 1 × TBST at room temperature for 1 hour. Detection of the protein signals was performed with enhanced chemiluminescence (MaestroGen, Hsinchu City, Taiwan) and the luminescence signal was detected using the UVP AutoChemi Image System. The western blot images were further quantified by ImageJ software (n = 3) and β–actin served for protein normalization in all groups.

### *In vitro* GCV sensitivity assay

Stable clone cells (1.5 × 10^4^) were seeded in 12-well cell culture plates. Cells were incubated for 12 hours for cell adhesion and treated with 0, 1, 5, or 10 μg/ml GCV for 5 days. After treatment, cells were placed in serum-free media containing 5 μg/ml MTT (Thiazolyl Blue Tetrazolium bromide; Sigma-Aldrich®, Cat. #M5655) and incubated at 37°C for 1 hour. The purple crystals formed by the cells were dissolved in dimethyl sulfoxide (DMSO; Sigma-Aldrich®, Cat. #D8418). The level of cell viability was determined by measuring the absorbance at 550 nm with an ELISA reader (ELx808; BioTek, Winooski, VT, USA).

### Staining for cell death

#### Propidium iodide (PI)/Hoechst 33342 co-staining

U251-pCMV-HSVtk or U251-pGFAP-HSVtk cells (1.5 × 10^4^) were seeded onto poly-L-lysine-coated 18-mm round coverslips in 12-well culture plates. After 12 hours of cell-adhesion, cells were treated with 5 μg/ml of GCV for 1, 3, 5, and 7 days. Treated cells were washed by 1 × DPBS and fixed using 4% paraformaldehyde (Sigma-Aldrich, Cat. #158127). Specimens were incubated in a nuclear dye of PI (Sigma-Aldrich, Cat. #4170)/Hoechst 33342 (*Thermo Fisher Scientific*, Cat. #62249) mixture diluted in 1 × PBS for 5 minutes, washed three times in 1 × PBS, and mounted with Fluoro-Gel (Electron Microscopy Sciences, Cat. #17985–10, Hatfield, PA, USA).

#### Terminal deoxynucleotidyl transferase dUTP nick end labeling (TUNEL) assay

The TUNEL assay was performed with a cell death detection kit (Roche Applied Science, Cat. # 11684795910) to detect DNA fragmentation generated during apoptosis. For cell apoptosis analysis, 1.5 × 10^4^ U251-pGFAP-HSVtk cells were treated with GCV for 3 days, as described. H_2_O_2_ (10 μM) was added to induce apoptosis in the positive control groups. All images were acquired using a DMR fluorescence microscope (Leica) and a LSM880 confocal microscope (Carl Zeiss, Oberkochen, Germany).

### Immunocytochemistry for nestin, GFAP, and βIII-tubulin

U251-pCMV-HSVtk or U251-pGFAP-HSVtk cells (1.5 × 10^4^) were seeded onto poly-L-lysine-coated 18-mm round coverslips in 12-well culture plates. After 12 hours of cell-adhesion, cells were treated with 5 μg/ml of GCV for 1, 3, 5, and 7 days. Cells were fixed with 100% methanol and 4% paraformaldehyde and blocked with 1% of FBS in phosphate buffer saline with 0.1% Triton X-100 (PBS-T) for 1 hour. Thereafter, cells were incubated with primary antibodies (see S1 Table) in blocking buffer at 4°C overnight. Cells were then incubated in fluorescent-labeled secondary antibodies (see S1 Table) for 1 hour at room temperature. Specimens were mounted with Fluoro-Gel (Electron Microscopy Sciences). Fluorescent images were acquired using a Carl Zeiss LSM880 confocal microscope. Photomicrographs were adjusted both in contract and brightness and merged using Photoshop software (Adobe, Mountain View, CA, USA, RRID: SCR_014199).

Survival rate and differentiation rate of U251-pGFAP-HSVtk and U251-pCMV-HSVtk were determined by quantifying five randomly selected fields of confocal images from each immunostaining. Survival rate were determined by calculating the number of Hoechst-positive cell nuclei on each treatment day compared with those on day 0. The ratio of nestin, GFAP, and βIII-tubulin positive cells in all viable cells (Hoechst-positive cells) were calculated as differentiation rates of U251-pGFAP-HSVtk and U251-pCMV-HSVtk.

### Establishment and analysis of recovery of U251-pCMV-HSVtk and U251-pGFAP-HSVtk subclones

After treatment with 5 μg/ml of GCV for 7 days, U251-pCMV-HSVtk and U251-pGFAP-HSVtk cells were supplemented with fresh growth medium for 3 days. The remaining cell populations were regarded as recovery subclones. Subsequently, PI/Hoechst 33342 staining was performed to investigate this cell population. In addition, immunocytochemistry of βIII-tubulin, GFAP, and nestin were conducted to study the expression patterns of neural markers in the recovery cell subclones.

### Statistical analysis

GraphPad Prism® 4.0 software (GraphPad Software, La Jolla, CA, USA, RRID: SCR_002798) was used to analyze the data. When comparing two conditions, a *t*-test was performed, with a *p* value < 0.05 regarded as statistically significant. All data were expressed as mean ± standard error of the mean (SEM).

## Results

### Plasmid design and the specificity of the CMV promoter and the GFAP promoter in various cell lines

HSVtk and P2A were subcloned into pEGFP-N3, and named as pCMV-HSVtk-P2A-EGFP, to serve as a positive control vector in which the HSVtk and the reporter gene EGFP were separately expressed (S1A Fig). P2A, a self-cleavage peptide, played a role similar to an IRES, but was shorter with higher cleavage efficiency. Therefore, the HSVtk and EGFP sequences could be cleaved after translation and would become individual proteins with the assistance of P2A. Based on the design of pCMV-HSVtk-P2A-EGFP, the experimental plasmid, pGFAP-HSVtk-P2A-EGFP, was cloned (S1B Fig). The CMV promoter was replaced by the GFAP promoter, the DNA for which was amplified from a pAAV-GFAP-hChR2(H134R)-mCherry plasmid and cloned into *AseI-XhoI* restriction sites using an In-Fusion cloning kit.

**Fig 1:**
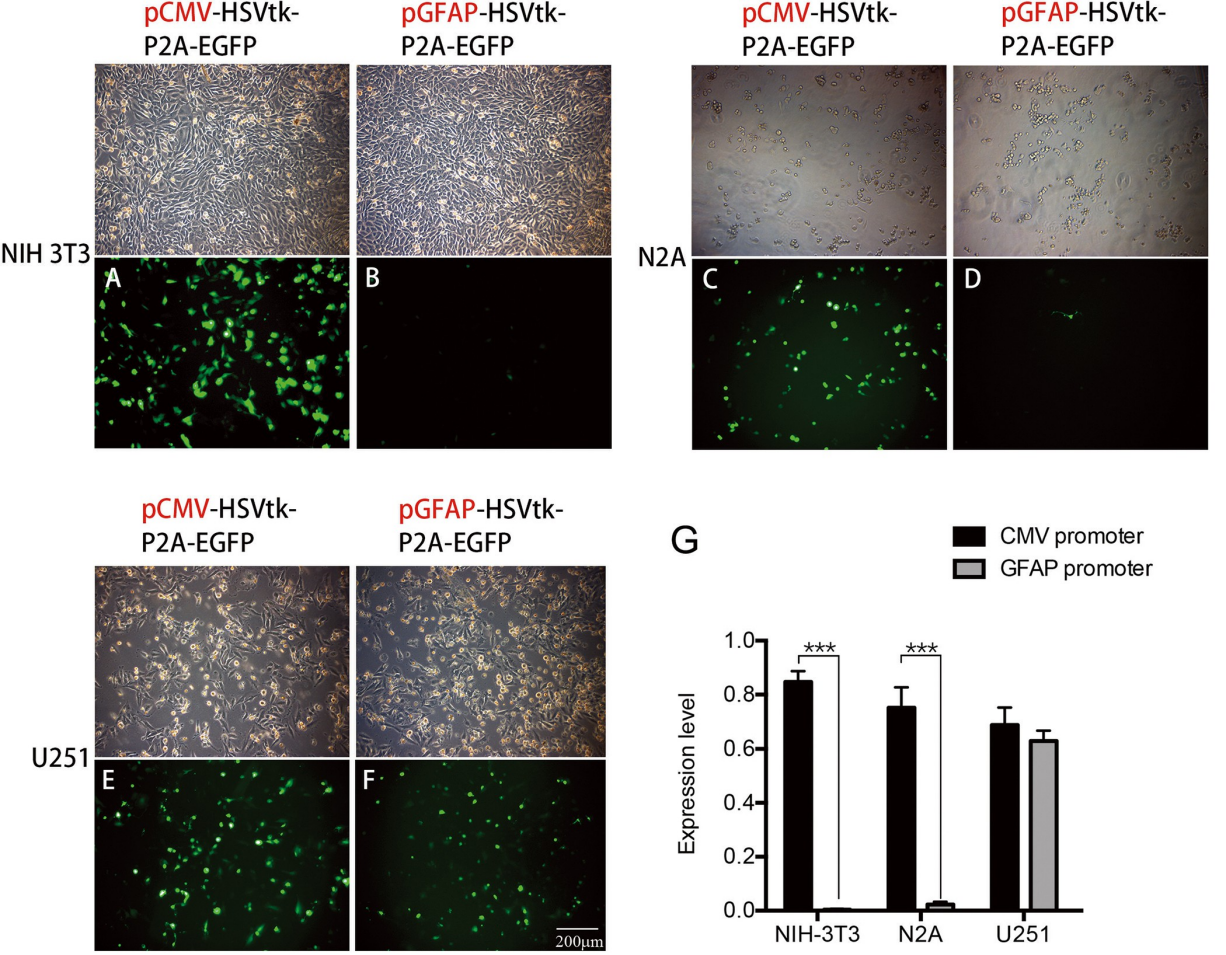
Expression levels of CMV and GFAP promoters in non-glial and glioblastoma cell lines. (A) Three cell lines—NIH-3T3, N2A, and U251—were transiently transfected with pCMV-HSVtk-P2A-EGFP or pGFAP-HSVtk-P2A-EGFP, and EGFP-positive cells were observed under an inverted fluorescence microscope. (B) NIH-3T3 had a strong expression level when transfected with the control plasmid, pCMV-HSVtk-P2A-EGFP. No NIH-3T3 cells expressed EGFP when transfected with pGFAP-HSVtk-P2A-EGFP. (C) A strong EGFP expression level was detected in N2A cells transfected with pCMV-HSVtk-P2A-EGFP. (D) Very few EGFP-positive cells were identified in N2A cells transfected with pGFAP-HSVtk-P2A-EGFP. Many EGFP-positive cells were observed in U251 cells transfected with pCMV-HSVtk-P2A-EGFP (E) or pGFAP-HSVtk-P2A-EGFP (F). Scale bar, 200 μm. (G) The proportion of EGFP-positive cells from all live cells was calculated as the expression level of the promotors (n = 5). There was no significant difference in the expression level of NIH-3T3, N2A, and U251 cell lines when transfected with pCMV-HSVtk-P2A-EGFP. By contrast, the expression level of pGFAP-HSVtk-P2A-EGFP was significantly higher in U251 cells than in NIH-3T3 or N2A cells.

To examine further the promoter specificity of pCMV-HSVtk-P2A-EGFP and pGFAP-HSVtk-P2A-EGFP in non-glial and glial cell lines, transient transfection into three cell lines—NIH-3T3 (mouse fibroblast cell line), N2A (mouse neuroblastoma cell line), and U251 (human glioblastoma cell line)—was conducted, and green fluorescent cells were analyzed on day 2 (Fig 1). A high proportion of cells containing green fluorescence were detected in all three cell lines transfected with pCMV-HSVtk-P2A-EGFP (Fig 1A, 1C and 1E). However, non-glial (NIH-3T3 and N2A) and glial (U251) cell lines displayed different expression levels after transfection with pGFAP-HSVtk-P2A-EGFP. Very few EGFP-positive cells were detected when NIH-3T3 and N2A cells were transfected with pGFAP-HSVtk-P2A-EGFP (Fig 1B and 1D). By contrast, many U251 cells expressed green fluorescence when transfected with pGFAP-HSVtk-P2A-EGFP (Fig 1F). All images were quantified by counting EGFP-positive cell numbers (Fig 1G). There was no significant difference in the number of EGFP-positive cells when pCMV-HSVtk-P2A-EGFP was transfected into the NIH-3T3, N2A, and U251 cell lines. However, after transfection with pGFAP-HSVtk-P2A-EGFP, the glioblastoma cell line, U251, had a higher proportion of green fluorescent cells (62.80±3.84%) than did NIH-3T3 and N2A cells (0.36±0.31% and 2.31±1.01% of EGFP-positive cells, respectively). These data on promoter specificity suggest that a large proportion of glia cells, but not neurons and fibroblasts, could be specifically targeted by a vector under the control of the GFAP promoter.

### Establishment of the stable clones and characterization of their HSVtk expression levels

To produce stable clones that constitutively expressed HSVtk, 500 μg/ml and 400 μg/ml of G418 were applied to N2A and U251, respectively. During 14 days of selection, cells with a strong EGFP signal formed colonies, which were isolated and amplified for further FACS enrichment. Cells that expressed strong green fluorescence were sorted and further enriched by cell sorting and regarded as candidates for stable clones. There was no significant difference in the morphology or cell doubling time between parent cells and the transfected cells used in this selection. We established four stable clones—N2A-pCMV-HSVtk, N2A-pGFAP-HSVtk, U251-pCMV-HSVtk, and U251-pGFAP-HSVtk—for use in subsequent experiments, as described below.

To verify the protein expression of these stable clones, the levels of HSVtk protein expression were determined by western blot (S2 Fig). Stable levels of cytosolic HSVtk protein were observed in N2A-pCMV-HSVtk cells within 4 weeks, while no protein was detected in N2A parent cells (S2A Fig). Since the GFAP promoter would not be driven in neuronal cells, no protein was detected from week 1 to week 4 in N2A-pGFAP-HSVtk cells (S2C Fig). By contrast, stable U251 clones displayed high-yielding HSVtk protein signals under CMV (S2B Fig) and GFAP (S2D Fig) promoter control. This examination of HSVtk expression levels indicated that the four stable clones were successfully established.

### *In vitro* GCV sensitivity assays for stable clones treated for 5 days

**Fig 2:**
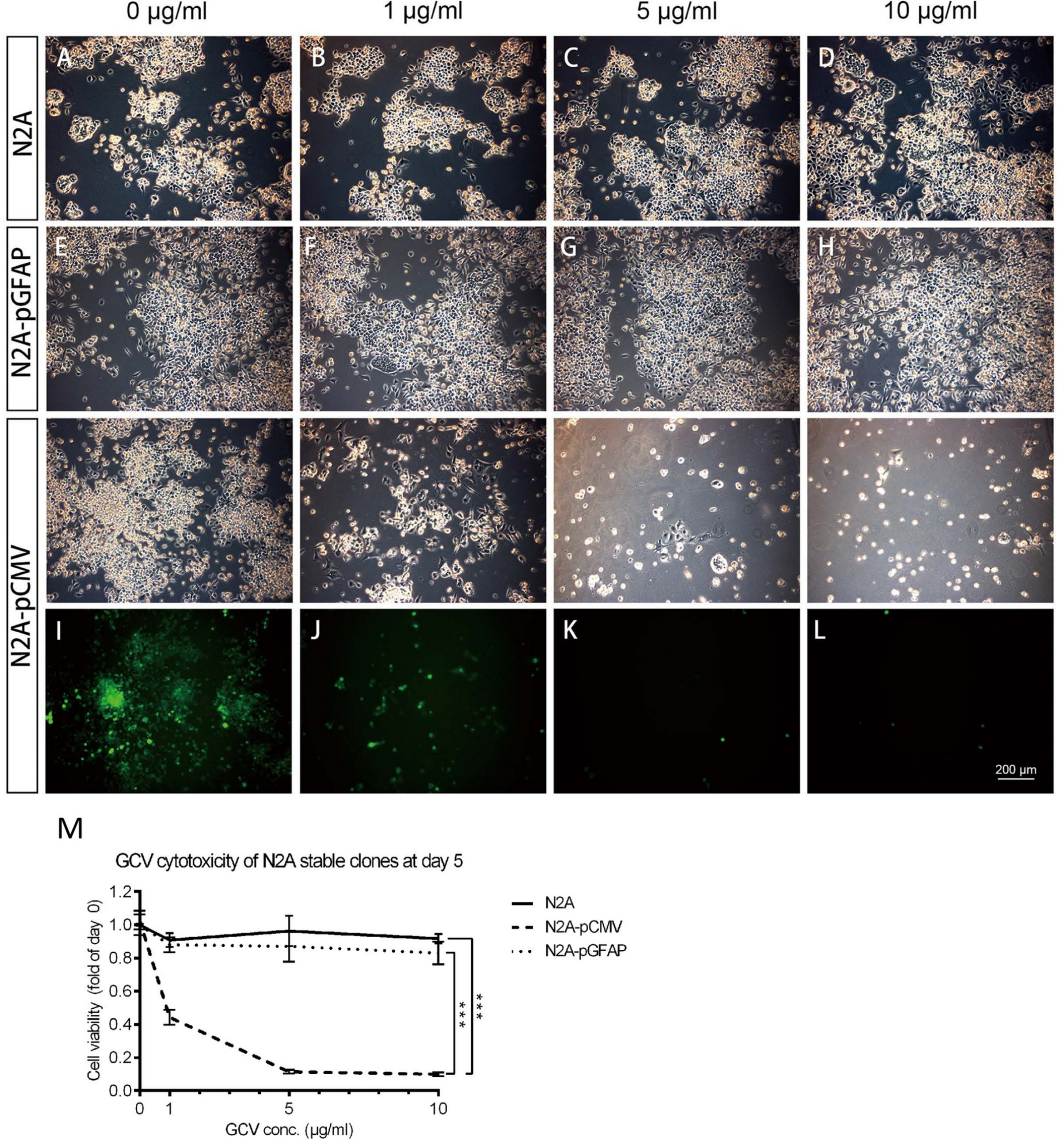
Sensitivity of N2A stable clones to GCV. (A–L) N2A, N2A-pGFAP-HSVtk, and N2A-pCMV-HSVtk cells were treated separately with 0, 1, 5, or 10 μg/ml of GCV for 5 days, and their live-cell phase images or fluorescent images were captured. (M) An MTT assay was performed to detect their cell viability under GCV challenge (n = 3). The morphology of N2A parent cells (A–D) and N2A-pGFAP-HSVtk cells (E–H) remained intact and their cell viability was maintained above 90%. (I–L) The number of N2A-pCMV-HSVtk cells was decreased in a dose-dependent manner. In addition, green fluorescent cells were still detectable among the surviving cells even after treatment with 10 μg/ml of GCV. (M) The results of the MTT assay for these three groups were consistent with the imaging results. Scale bar, 200 μm.

To investigate the sensitivity of the stable clones to GCV and determine the optimal working concentration for all cell lines, the cell viabilities in different GCV concentrations were tested. All stable clones were treated with 0, 1, 5, or 10 μg/ml GCV for 5 days, and then an MTT assay was performed to evaluate cell viabilities. Live-cell phase images and immunocytochemistry were used to examine cell morphology and EGFP expression. Phase images collected using an inverted microscope revealed that N2A parent cells (Fig 2A–2D) and transfected N2A-pGFAP-HSVtk cells (Fig 2E–2H) remained intact and maintained more than 90% cell viability. N2A-pCMV-HSVtk was the only group in which the cell number decreased in a dose-dependent manner (Fig 2I–2L). Furthermore, green fluorescence was still detectable in the surviving cells, even after treatment with 10 μg/ml GCV. Similar results were obtained from the MTT assay analysis, and N2A-pCMV-HSVtk was the only stable clone affected by GCV, with only 9.90±1.04% of the cells remaining after 10 μg/ml GCV treatment (Fig 2M). These results indicate that only N2A-pCMV-HSVtk cells were sensitive to GCV, since N2A parent cells and transfected N2A-pGFAP-HSVtk cells do not express HSVtk.

**Fig 3:**
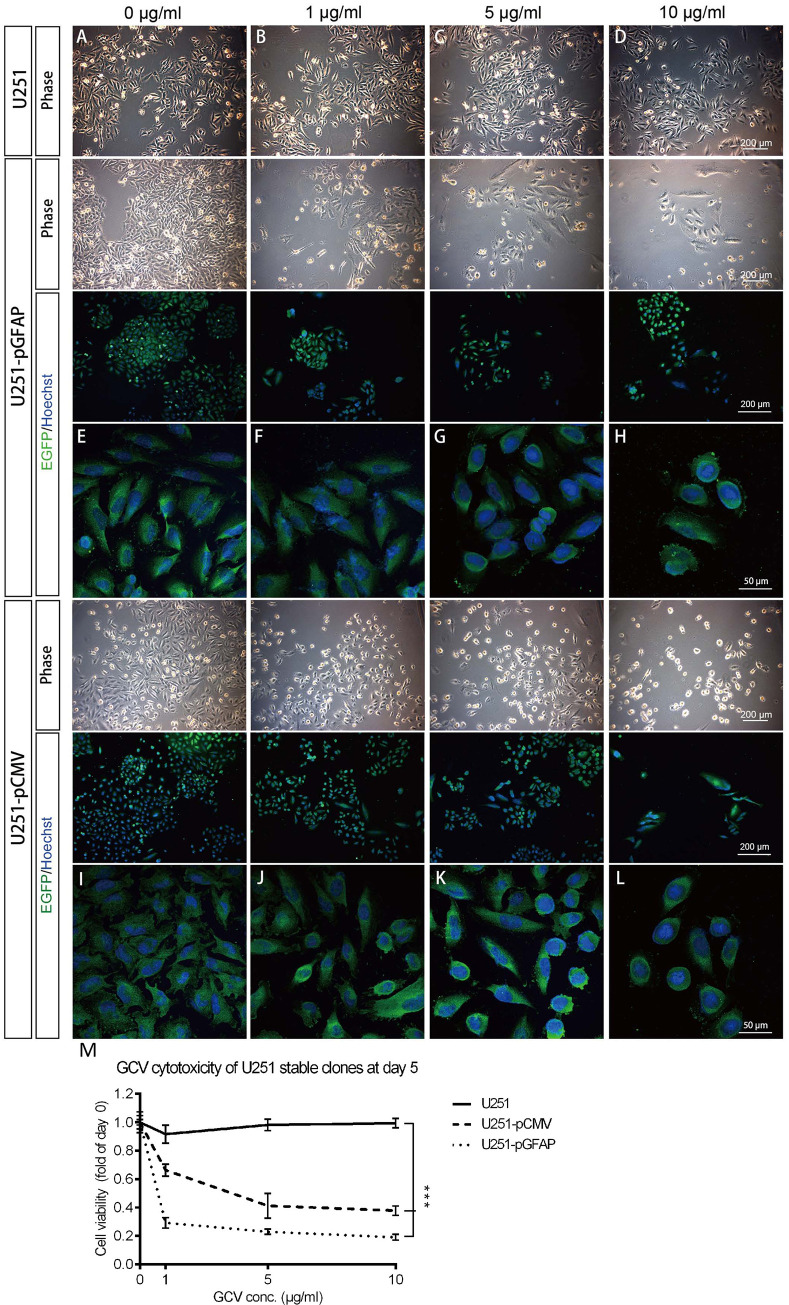
Sensitivity of U251 stable clones to GCV. U251, U251-pCMV-HSVtk, and U251-pGFAP-HSVtk cells were treated with 0, 1, 5, or10 μg/ml of GCV for 5 days. (A–D) Live-cell phase images of U251 parent cells displayed no cytotoxicity up to 10 μg/ml of GCV. (E–H) Live-cell phase images and confocal images of immunocytochemistry of U251-pGFAP-HSVtk revealed decreased cell numbers as the GCV concentration increased. (I–L) Live-cell phase images and confocal images of immunocytochemistry of the positive control cell clone, U251-pCMV-HSVtk, revealed a similar pattern with U251-pGFAP-HSVtk. (H, L) Some green fluorescent cells were detected to have undergone cell shrinkage when U251-pGFAP-HSVtk and U251-pCMV-HSVtk cells were treated with 10 μg/ml of GCV. (M) Cell viability detected by MTT assay (n = 3) demonstrated that both U251-pGFAP-HSVtk and U251-pCMV-HSVtk cells were sensitive to GCV. Scale bars, 200 μm or 50 μm, as indicated.

U251 parent cells sustained their proliferation in the presence of treatment with different concentrations of GCV (Fig 3A–3D). The number of both U251-pGFAP-HSVtk cells (Fig 3E–3H) and U251-pCMV-HSVtk cells (Fig 3I–3L) decreased in a dose-dependent manner as the GCV concentration increased. In spite of decreasing cell number, all remaining cells expressed EGFP and gradually rounded-up at higher GCV dosages (Fig 3H and 3L). The MTT assay analysis demonstrated that the cell viability of both U251-pGFAP-HSVtk (22.92±1.90%) and U251-pCMV-HSVtk (41.24±8.74%) cells after 5 μg/ml GCV treatment decreased to about 23% and 42%, respectively, of the control group not subjected to GCV treatment (98.13±4.02%) (Fig 3M), indicating that both U251-pGFAP-HSVtk and U251-pCMV-HSVtk cells were sensitive to GCV. Furthermore, U251-pCMV-HSVtk cells, as a control for all the stable clones, reached LD_50_ at 5 μg/ml GCV treatment. Therefore, the optimal dosage of GCV chosen for ablating stable clones in subsequent experiments was 5 μg/ml.

In summary, the experimental stable clone, U251-pGFAP-HSVtk, and the positive-control stable clone, U251-pCMV-HSVtk, displayed a dose-dependent response to GCV, while only N2A-pCMV-HSVtk was sensitive to GCV. N2A-pGFAP-HSVtk cells remained intact because the GFAP promoter would not be expressed in N2A neuronal cells. These data demonstrate that the GFAP promoter can specifically drive the HSVtk/GCV system and selectively ablate glioblastoma cell lines, while keeping neuronal cell lines intact.

### Cell apoptosis assays and cell viability assay after GCV treatment

A previous study demonstrated that the HSVtk/GCV system triggered cells to undergo apoptosis rather than necrosis [29]. Therefore, we investigated whether the apoptotic pathway was activated in the cell lines treated with GCV and performed the TUNEL assay which can identify DNA nicks resulting from apoptotic signaling cascades (S3 Fig). Only cells that were both TUNEL- and Hoechst-positive, displayed condensed nuclei, and were magenta-colored in merged confocal images were considered apoptotic cells. In the GCV-treated group, apoptotic cell nuclei with TUNEL- and Hoechst-positive signals were identified (S3A–S3C Fig) and displayed a rounded and bulging morphology in differential interference contrast (DIC) images (S3D Fig). All cell nuclei in the positive control group were TUNEL- and Hoechst-positive (S3G Fig), whereas those in the negative control group displayed only Hoechst staining (S3K Fig). These results further indicate that the death of U251-pGFAP-HSVtk cells induced by 3 days of GCV treatment was via apoptosis.

To identify dead cells and evaluate cell viability, U251-pCMV-HSVtk and U251-pGFAP-HSVtk cells treated with GCV for 7 days were subjected to PI/Hoechst double nuclear staining (S4 Fig). Very weak PI signals appeared in U251-pGFAP-HSVtk cells on day 3 (S4C Fig), while cells with strong PI signals were detected on days 5 and 7 (S4D and S4E Fig). The condensed nuclei of these cells displayed double-positive PI and Hoechst staining (S4D and S4E Fig, arrows). PI signals were undetectable after GCV was withdrawn for 3 days (S4F Fig). The control cell line, U251-pCMV-HSVtk, displayed similar results, with weak PI signals by day 3 (S4I Fig) and stronger signals from day 5 to 7 (S4J and S4K Fig). No PI signals were detected in cell nuclei after recovery (S4L Fig). In summary, U251-pGFAP-HSVtk cells underwent apoptosis on day 3 and cells in a late apoptotic phase were identified by day 5 of GCV treatment.

### Pattern of nestin, GFAP, and βIII-tubulin expression in U251 stable clones after GCV treatment

**Fig 4:**
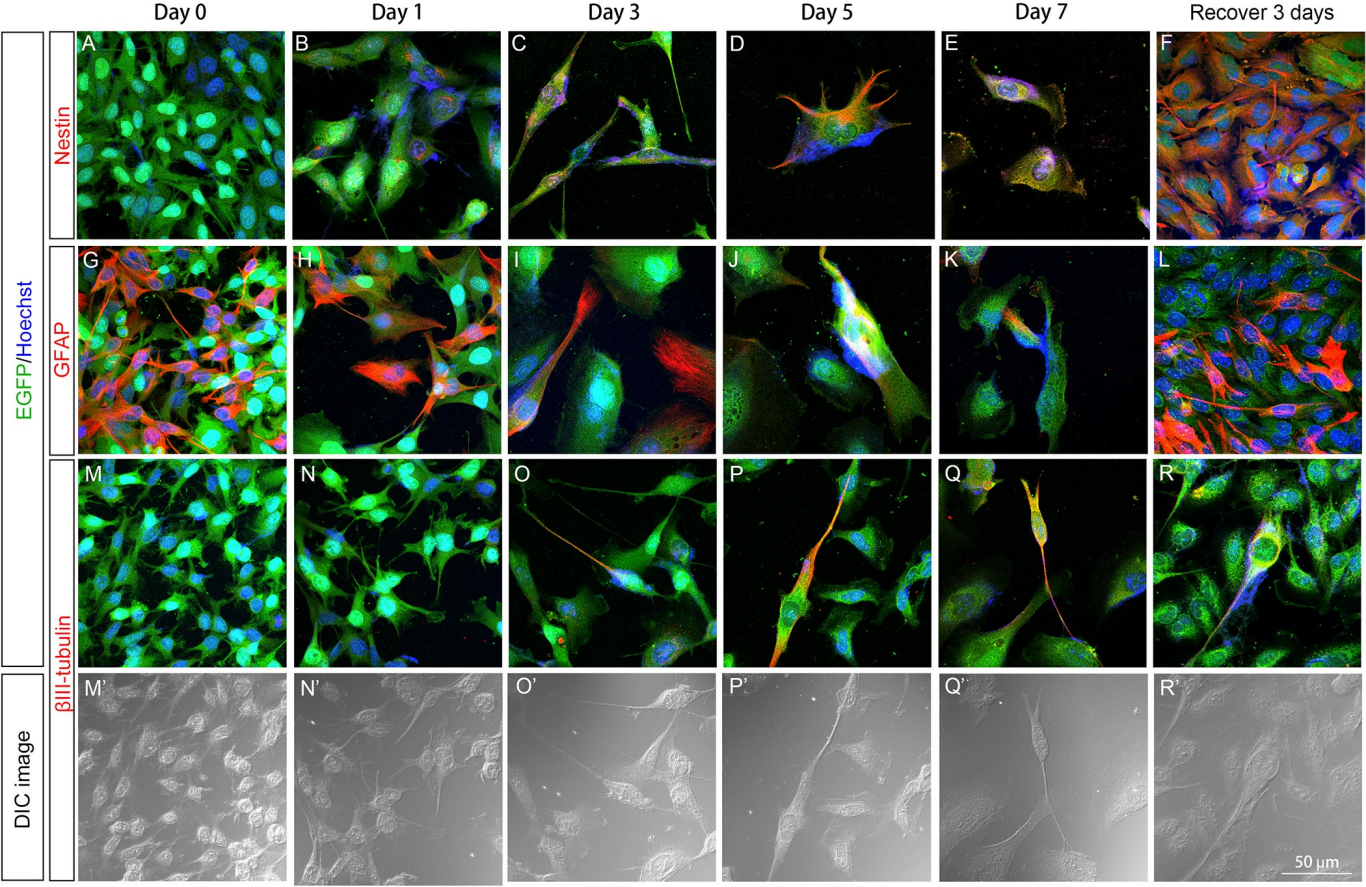
Patterns of nestin, GFAP, and βIII-tubulin expression in U251-pGFAP-HSVtk cells. U251-pGFAP-HSVtk cells were treated with 5 μg/ml GCV for 7 days, and then GCV was withdrawn to allow cell recovery for 3 days. Immunocytochemistry of nestin (A–F), GFAP (G–L), and βIII-tubulin (M–R, M′–R′) were performed to characterize the identity of surviving cells. The expression level of nestin was increased during 7 days of GCV treatment (A–E) and strong expression of nestin was detected after recovery (F). (D–F) Filamentous expression of nestin was detected at day 5, day 7, and after recovery. (H–K) U251-pGFAP-HSVtk cells with strong GFAP expression at day 0 (G) were gradually ablated during 7 days of GCV treatment. (L) After GCV was withdrawn for 3 days, some cells expressed a high level of GFAP. (G–L) Filamentous GFAP could be found in cell processes and cell bodies. βIII-tubulin could not be detected at day 0 and day 1 (M, N), and was observed weakly at day 3 of GCV treatment (O). (P–R) βIII-tubulin-positive cells were identified after 5 days of GCV treatment and recovery for 3 days. (M′–R′) In DIC images, βIII-tubulin-positive cells displayed elongated processes, rounded nuclei, and bulging cell bodies, while βIII-tubulin-negative cells were smooth. Scale bar, 50 μm.

**Fig 5:**
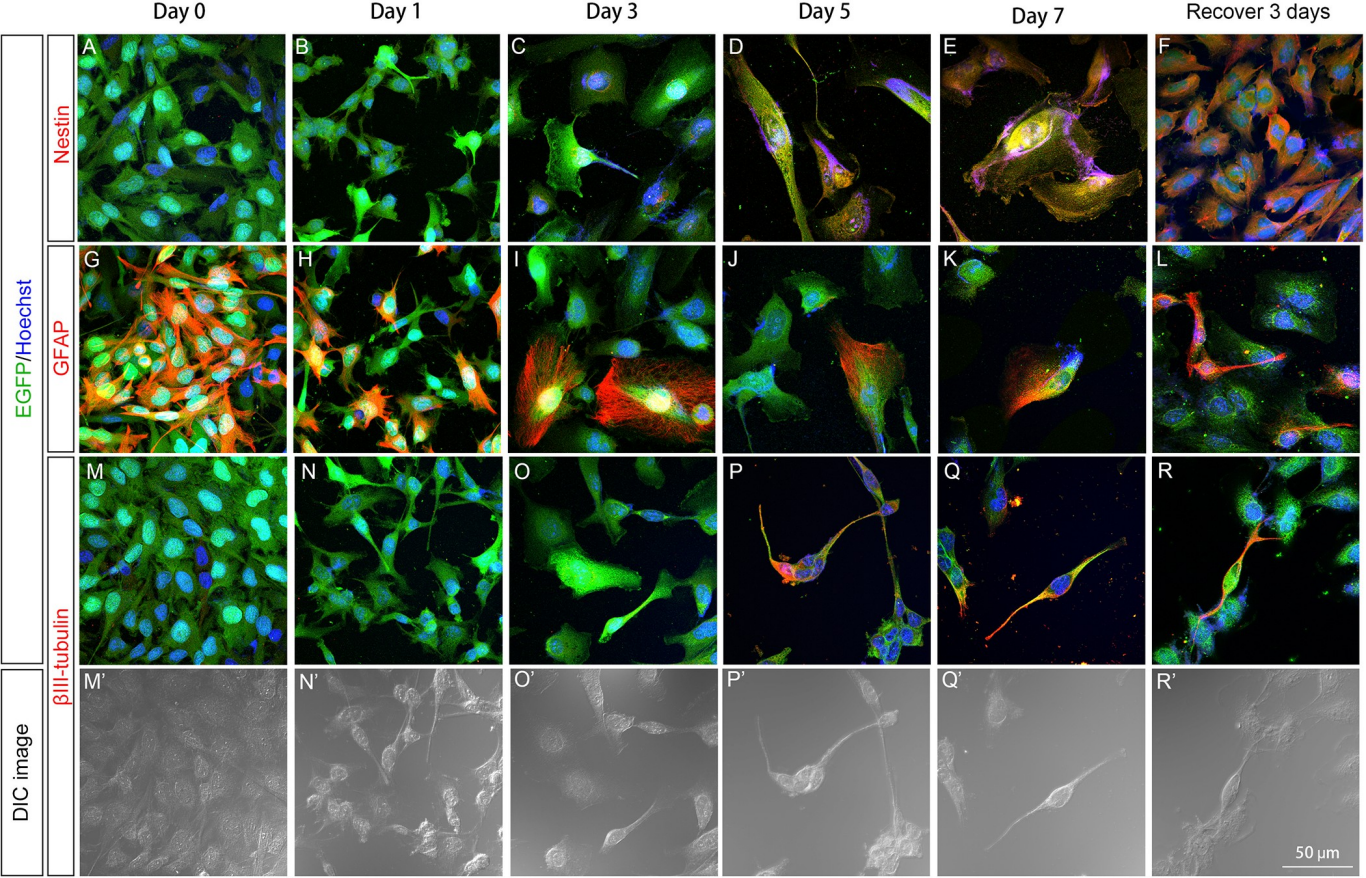
Patterns of nestin, GFAP, and βIII-tubulin expression in U251-pCMV-HSVtk cells. U251-pCMV-HSVtk cells were treated with 5 μg/ml GCV for 7 days, and then GCV was withdrawn to allow for cell recovery for 3 days. Immunocytochemistry of nestin (A–F), GFAP (G–L), and βIII-tubulin (M–R, M′–R′) was performed to characterize the identity of surviving cells. Increasing levels of nestin expression were detected during 7 days of GCV treatment (A–E), and strong expression of nestin was detected after recovery (F). (D–F) Filamentous expression of nestin was observed at day 5, day 7, and after recovery. U251-pCMV-HSVtk cells expressed GFAP strongly at day 0 (G), and then gradually decreased expression during 7 days of GCV treatment (H–K). (L) After recovery for 3 days, increased expression of GFAP was observed in U251-pGFAP-HSVtk cells. (G–L) Filamentous GFAP expression was observed in cell processes and cell bodies. (M–R) βIII-tubulin positive neuron-like cells could not be detected until day 5 of GCV treatment and after recovery for 3 days. (M′–R′) DIC images also revealed that βIII-tubulin-positive cells had elongated processes, rounded cell nuclei, and bulging cell bodies, while other cells were smooth. Scale bar, 50 μm.

Enlargement and process extension of surviving (or survival) cells, which are morphological features of cell differentiation, were observed in U251-pGFAP-HSVtk and U251-pCMV-HSVtk cells during GCV treatment. Therefore, immunocytochemical staining for nestin, GFAP, and βIII-tubulin was performed to characterize the identity of the survival cells in the U251-pGFAP-HSVtk (Fig 4) and U251-pCMV-HSVtk (Fig 5) groups. In addition, cells in recovery subclones were also investigated to mimic recurrence after drug withdrawal. Nestin, a stem cell marker, was used to identify neural stem cells. Only a trace of nestin signal was detected on day 1 (Fig 4B) of GCV treatment, but cells with filamentous nestin expression were detected from day 3 to day 7 (Fig 4C–4E) in U251-pGFAP-HSVtk cells. After 3 days of recovery, recovered U251-pGFAP-HSVtk cells had strong nestin expression (Fig 4F). These results indicate that cells with stem cell properties had more resistance to GCV and this population of neural stem-like cells was enriched after GCV withdrawn. The expression of GFAP in U251-pCMV-HSVtk cells gradually decreased during the 7 days of GCV treatment (Fig 4G–4K), suggesting that cells with strong GFAP expression were eliminated by GCV treatment. However, cells with weak GFAP expression might survive and resume proliferation after GCV withdrawal; as a result, more cells with strong GFAP expression were observed in the recovered cell population (Fig 4L). These results also confirmed that GFAP-positive glioblastoma cells could be ablated by the HSVtk/GCV system controlled by the GFAP promoter.

For identification of neuronal differentiation, the expression of βIII-tubulin, a neuronal marker, was investigated. Weak βIII-tubulin expression was detected by day 3 of GCV treatment (Fig 4O), and survival cells with strong βIII-tubulin expression in their processes were detected on days 5 and 7 and in the recovery cell population (Fig 4P–4R). According to DIC images, these βIII-tubulin-positive cells had elongated processes, rounded cell nuclei, and bulging cell bodies, while other cells were smooth (Fig 4P′–4R′). This βIII-tubulin expression indicated that neuron-like cells could be identified after 5 days of GCV treatment, and these cells remained after recovery. In summary, U251-pGFAP-HSVtk cells had the ability to undergo neural differentiation under the stress of GCV, and the recovered cells were enriched with neural stem-like cells.

U251-pCMV-HSVtk cells, which served as a positive-control, stable clone for U251-pGFAP-HSVtk cells, were also immunostained for nestin, GFAP, and βIII-tubulin (Fig 5). Similar patterns of nestin, GFAP, and βIII-tubulin expression were identified in U251-pCMV-HSVtk cells treated with GCV. Nestin-positive cells displayed better survivability during GCV treatment (Fig 5D and 5E), and a neural stem-like subclone with strong nestin expression was also enriched after recovery (Fig 5F). Decreasing GFAP expression was observed in survival cells from day 1 to day 7 of GCV treatment (Fig 5G–5K). Neuron-like cells, which were βIII-tubulin-positive, were identified after day 5 of GCV treatment and after GCV withdrawal (Fig 5P–5R). These βIII-tubulin-positive cells also had elongated processes, rounded cell nuclei, and bulging cell bodies, while other cells were smooth (Fig 5P′–5R′).

**Fig 6:**
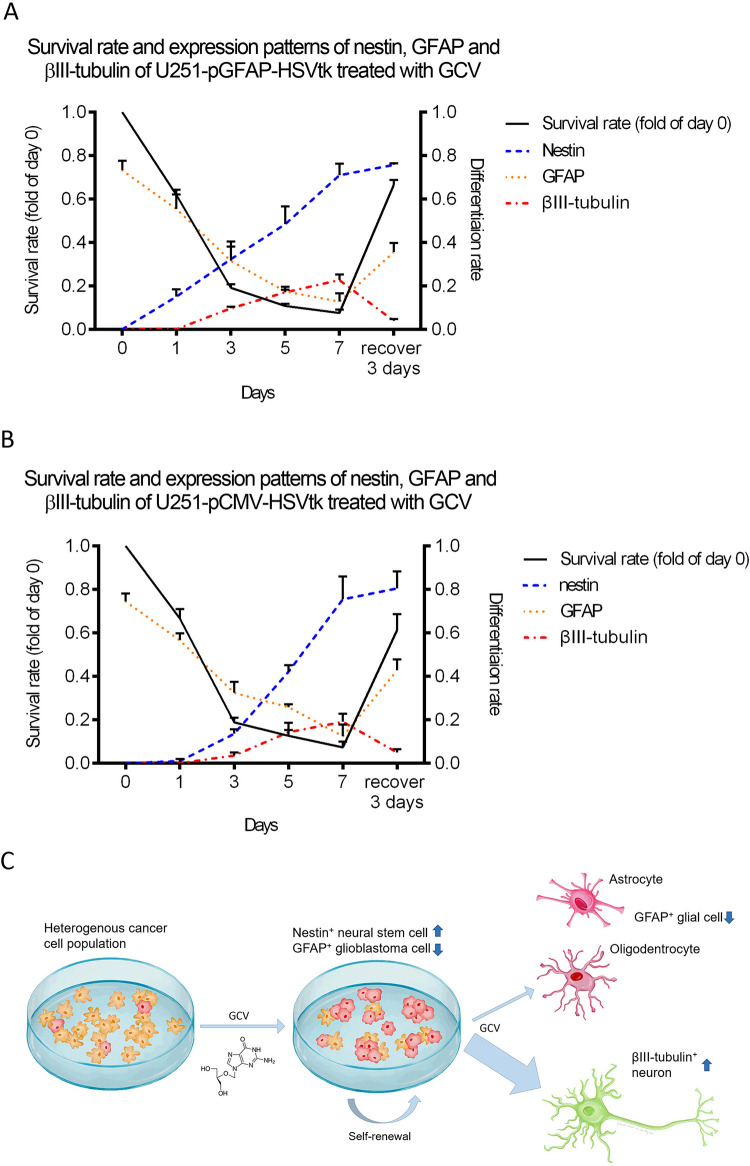
Summary of the main results in this study. The survival rate and expression ratio of nestin, GFAP, and βIII-tubulin in U251-pGFAP-HSVtk (A) and U251-pCMV-HSVtk (B) cells were determined by quantifying confocal images of immunocytochemistry (n = 3). The survival rate of both stable clones was decreased when cells were treated with GCV, but increased after recovery. The ratio of nestin-positive cells in both clones increased during GCV treatment and even after recovery. The ratio of GFAP-positive cells in both clones decreased during GCV treatment and increased after recovery. The ratio of βIII-tubulin-positive cells in both clones increased during GCV treatment and decreased after recovery. (C) The HSVtk/GCV system driven by the GFAP promotor could successfully ablate GFAP-positive glioblastoma cells while leaving non-glial cells intact. In addition, neural differentiation was promoted after challenge with this HSVtk/GCV system.

The survival rates and the expression ratio of the nestin-, GFAP-, and βIII-tubulin-positive cells in the U251-pGFAP-HSVtk (Fig 6A) and U251-pCMV-HSVtk (Fig 6B) cell groups were also determined by quantifying confocal images of the respective immunocytochemistry. Survival rates were determined by calculating the number of Hoechst-positive cell nuclei on each day of treatment compared with day 0 (without GCV treatment). The ratio of the nestin-, GFAP-, and βIII-tubulin-positive cells to all viable cells (Hoechst-positive cells) were calculated in U251-pGFAP-HSVtk and U251-pCMV-HSVtk cell groups during the 7 days of GCV treatment (Table 1). The survival rate of both stable clones gradually decreased during GCV treatment, but increased after GCV was withdrawn. Nestin-positive U251-pGFAP-HSVtk and U251-pCMV-HSVtk cells increased during the GCV treatment and even after GCV withdrawal. GFAP-positive U251-pGFAP-HSVtk and U251-pCMV-HSVtk cells decreased during GCV treatment and increased after 3 days of recovery. The βIII-tubulin-positive cells increased during GCV treatment and decreased after recovery. In summary, based on these results with U251-pGFAP-HSVtk and U251-pCMV-HSVtk cells, we suggest that neural stem-like cells with nestin expression are enriched after GCV treatment. Furthermore, a rapid decrease in the GFAP-positive cell number was observed during the 5 days of treatment. Finally, βIII-tubulin-positive neuron-like cells were detected after 3 days of GCV treatment.

**Table 1:** Summary of differentiation rate of βIII-tubulin, GFAP, and nestin during GCV treatment.

	**U251-pGFAP-HSVtk**					
Day(s)	0	1	3	5	7	Recover 3 days
βIII-tubulin	0	0	0.09±0.01	0.17±0.02	0.23±0.03	0.04±0.01
GFAP	0.73±0.04	0.55±0.07	0.32±0.09	0.17±0.01	0.13±0.04	0.36±0.04
Nestin	0	0.15±0.03	0.32±0.06	0.48±0.08	0.71±0.05	0.76±0.01
	**U251-pCMV-HSVtk**					
Day(s)	0	1	3	5	7	Recover 3 days
βIII-tubulin	0	0	0.04±0.01	0.14±0.04	0.19±0.04	0.05±0.01
GFAP	0.74±0.04	0.57±0.03	0.32±0.05	0.26±0.01	0.12±0.05	0.43±0.05
Nestin	0	0.01±0.01	0.14±0.02	0.42±0.03	0.75±0.10	0.80±0.01

Together, these studies have demonstrated that GCV can promote neural differentiation of U251-pGFAP-HSVtk and U251-pCMV-HSVtk cells. In addition, we propose that the HSVtk/GCV system can be used to enrich neural stem-like cells and eliminate glioblastoma *in vitro*, while survival cells can differentiate into neuron-like cells.

## Discussion

In this study, the GFAP promoter, an astrocyte-specific promoter, was used to restrict HSVtk gene expression in the glioblastoma cell line, U251. The specificity of the GFAP promoter in pGFAP-HSVtk-P2A-EGFP was confirmed by comparison with the control plasmid, pCMV-HSVtk-P2A-EGFP. An extremely low number of EGFP-positive cells was detected when NIH-3T3 and N2A cells were transfected with pGFAP-HSVtk-P2A-EGFP. A previous study demonstrated that a different GFAP promoter could be expressed in various kinds of cell lines, and the expression level of the GFAP promoter was 1.3% and 3.5% in N2A and NIH-3T3 cell lines, respectively [30]. In addition, gene regulation studies have indicated that the promoter and its distal elements could engage in looping interactions [31]. Long-range interactions between promoters and distal sites, including elements resembling enhancers, promoters, and CCCTC-binding factor (CTCF)-bound sites interacted with elements located ~120 kb upstream of the transcription start sites [32]. Furthermore, mammalian cells possess a high activity of illegitimate recombination. When DNA is transfected into mammalian cells, illegitimate recombination is a predominant pathway of integration of transfected DNA into the host genome [33, 34]. Thus, some other gene regulators that are located more than several hundred bp upstream from the GFAP promoter may exist, and may exhibit long-range interaction with the HSVtk gene, which was integrated into the host genome randomly. These issues may be the causes of the leaking problem found in NIH-3T3 and N2A cells.

In this research, the GFAP promoter was applied to control the expression of the HSVtk gene in glioblastoma cell lines, while the cytotoxicity of GCV was limited in neurons and fibroblasts. Our result could correspond to the previous study which showed that the HSV-TK gene driven by human GFAP promoter could selectively kill C6 and U251 glioma cell lines, with no effect on the ovarian cells [28]. Besides, the efficacy of HSV-TK gene-mediated cell killing was also reported in the primary cultures of human glioblastomas, which the majority of the tumor cells had detached or showed cytopathic effects after four-day GCV treatment [19]. These results suggested that HSVtk/GCV system could be applied to glioblastoma research. In addition, the HSVtk/GCV system was also driven by other promoters in previous studies. The CMV promoter is a constitutive mammalian promoter [35]. Bone marrow mesenchymal stem cells (MSCs) transduced with a recombinant baculovirus vector containing the HSVtk gene driven by the CMV promoter were reported to act as a systemic cancer therapy. The transduced MSCs were able to deliver the HSVtk suicide gene to glioblastoma and inhibited tumor growth on introduction of GCV [36]. Transcriptional targeting promoters were also used to mediate HSVtk expression in order to selectively target tumors [37]. Prostate-specific antigen (PSA) promoter was another tissue-specific promoter used in targeting prostate cancer [38]. Because telomerase is highly active in human cancer cells, the promoter regions of the telomerase RNA and catalytic component, human telomerase reverse transcriptase, have been used in many targeted cancer gene therapies to drive therapeutic gene expression [39]. Glucose-regulated protein 78 (Grp78) plays an essential role in cancer cell survival, so the Grp78 promoter along with HSVtk expression was applied to infect tumor cells for cancer suicide therapy [40]. In the present study, we confirmed that the GFAP promoter could specifically control HSVtk expression in glioblastoma cell lines while leaving neuronal cell lines intact. Moreover, we demonstrated that a glioblastoma cell line with a high level of GFAP expression could be eliminated on day 7 of GCV treatment.

Our results also demonstrated that the HSVtk/GCV system promoted neural differentiation of U251 cells and enriched the neural stem-like cell population after 7 days of GCV treatment. We demonstrated that cells with a high GFAP expression level were killed, while βIII-tubulin-positive neuron-like cells were identified after day 5 of GCV treatment. Previous studies have also revealed that some drugs can induce neural differentiation in other glioblastoma cell lines. The novel small molecule, CG500354, was reported to induce growth arrest of GBM-derived cells and neural differentiation via the cAMP/CREB signaling pathway. In contrast to our results, CG500354 treatment decreased the number of nestin-positive neural progenitor cells and increased the number of GFAP- and Tuj1-(βIII-tubulin)-positive neural cell types [41]. Furthermore, rapamycin, a therapeutic agent with both immunosuppressant and antitumor properties [42], reduced the proliferation of glioblastoma cells and increased the expression of neural differentiation markers (GFAP and βIII-tubulin) [12]. Another natural product, curcumin, has a potent anticancer effect on a variety of cancer cell types. Curcumin activated autophagy and triggered the differentiation cascade of human glioblastoma by producing higher levels of βIII-tubulin and GFAP expression [13]. Therefore, our finding that the HSVtk/GCV system driven by the GFAP promotor promoted the neural differentiation of U251 cells displayed a similar effect to other reports.

To test the HSVtk/GCV system on another genomic background, another human primary glioblastoma cell line, U87, was examined in our laboratory (unpublished data). The HSVtk/GCV system driven by the GFAP promoter could ablate GFAP-positive glioblastoma cell lines and promote neural differentiation within 5 days of GCV treatment. A similar trend found in U87 cells indicated that this system represents a promising preclinical test for glioblastoma.

In this study, we showed that cell number of GFAP-positive cells would decrease during the treatment of GCV, and increase after 3 days of GCV withdraw. We demonstrated that GFAP promoter could specifically drive the HSVtk/GCV system and selectively ablate GFAP-positive cells. One reason for the increase of GFAP-positive cells after 3 days recovery may be due to the differentiation of the nestin-positive neural stem cell. Previous studies reported that cancer stem cells could be identified in U251 glioma cell line [43, 44] and cancer stem cells represented a population of drug-resistant cells that could survive treatment and repopulate the tumor. Besides, side populations of glioblastoma cells (U251 and primary glioblastoma sample) were less sensitive to HSV-TK/GCV system than the non-side population [45], indicating that GFAP-positive cells may not be fully ablate under GCV treatment in our study. Therefore, the second reason for the increase of GFAP-positive cells after 3 days recovery may come from the proliferation of surviving GFAP-positive cells.

We observed that the nestin-positive neural stem cell increased after the GCV treatment and βIII-tubulin-positive neuron-like cells gradually increased after 5 days of GCV treatment and decreased after 3 days recovery. We suggested that the increase of βIII-tubulin-positive neuron-like cells during GCV treatment may be further differentiated from nestin-positive neural stem cell which increased from day 3 of GCV treatment. Because previous study suggested that cellular origin may affect lineage differentiation propensity of human induced pluripotent stem cells [46], nestin-positive neural stem cell may tend to differentiate into GFAP-positive cells instead of neurons after GCV was withdrawn. As a result, βIII-tubulin-positive neuron-like cells decreased after 3 days recovery. In this study, we did not investigate the maturation of βIII-tubulin-positive neuron-like cells, so further neuronal markers could be applied to identify the cell property in the future.

The HSVtk/GCV system can cause the death not only of HSVtk-positive cells but also of the surrounding HSVtk-negative tumor cells, which is called bystander effect and is another important feature of this system [47]. Bystander effect is critical for the clinical application because gene transfer currently available typically result in fewer than 10% of the cells expressing the transgene [48]. Previous study suggested that gap junction communication was the mediator of the bystander effect in primary cultures of human glioblastoma cells by transferring phosphorylated GCV from HSVtk-transfected cells to untransfected ones [49]. U251 glioblastoma cell line was shown to exhibit high gap junctional intercellular communication between neighboring cells (>80%) [50] and U251 cells expressing HSVtk gene could induce cytotoxicity in cocultured U251 bystander cells that lack the viral kinase when incubated with GCV [51]. In this study, the bystander effect has not been evaluated and further study could investigate the difference of the bystander effect between U251-pGFAP-HSVtk and U251-pCMV-HSVtk cells and the differentiation ability of the bystander tumor cells induced by bystander effect.

While the precise mechanisms underlying the neural differentiation are not known, some possible mechanisms are suggested by other studies. Autophagy is involved in differentiation, survival, and cell death, and the PI3K/AKT/mTOR pathway was reported to promote growth and proliferation over differentiation of adult stem cells, and neural stem cells, specifically [52]. Inactivation of the mTOR pathway was sufficient to induce autophagy following the administration of rapamycin [12]. Furthermore, curcumin promoted differentiation by inducing autophagy [13]. Therefore, it could be speculated that the HSVtk/GCV system promotes neural differentiation of glioblastoma, and this is mediated by the PI3K/AKT/mTOR-related pathway. However, evidence for the involvement of a specific signaling pathway should be further investigated in future studies.

In conclusion, this study established pGFAP-HSVtk-P2A-EGFP plasmids that could successfully ablate GFAP-positive glioblastoma cell lines, while neuronal N2A cells remained intact. The data also suggested that the neural differentiation of glioblastoma cells could be promoted after challenge with the HSVtk/GCV system.

## Conclusions

This study constructed a pGFAP-HSVtk-P2A-EGFP plasmid bearing the HSVtk/GCV system controlled by the GFAP promoter to establish a strategy for cancer management *in vitro*. We demonstrated that this system could selectively ablate GFAP-positive glioblastoma cell lines, while it left the neuronal N2A cells intact and promoted neural differentiation after challenge with the HSVtk/GCV system (Fig 6C). Therefore, we propose that this system has potential for use in the study of the neural differentiation of glioblastoma stem cells *in vitro* and *in vivo* and offers a possible preclinical test for glioblastoma.

## Supporting information

S1 Fig Plasmid map of constructed pCMV-HSVtk-P2A-EGFP and pGFAP- 3 HSVtk-P2A-EGFP 4. (A) HSVtk (1131 bps) gene was subcloned into pEGFP-N3 at *XhoI* and *EcoRI* cutting sites. A 66 bps self-cleavage peptide P2A was also subcloned into pEGFP-N3 at *EcoRI* and *SalI* restriction enzyme cutting sites to obtain a construct named pCMV-HSVtk-P2A-EGFP. This construct could express HSVtk and EGFP as a reporter gene separately. Kanamycin/neomycin resistance gene was used for both kanamycin selection of *E*.*coli* colony and G418 sulfate selection of cell stable clone. (B) CMV promoter of pCMV-HSVtk-P2A-EGFP was substituted by 2207 bps GFAP promoter from another plasmid, pAAV-GFAP-hChR2(H134R)-mCherry, at restriction enzyme cutting sites of *AseI* and *XhoI*. HSVtk and EGFP in this clone were controlled by GFAP promoter, which is a specific promoter of glia cell. Kanamycin/neomycin resistance gene was used for both kanamycin selection of *E*.*coli* colony and G418 sulfate selection of stable clone in the further experiments.
(TIF)

S2 Fig HSVtk protein expression profile of stable clones by western blot. HSVtk protein expression levels of 4 stable clones, N2A-pCMV-HSVtk, U251-pCMV-HSVtk, N2A- pGFAP-HSVtk, and U251-pGFAP-HSVtk, at the 1^st^, 2^nd^, and 4^th^ week were demonstrated by western blot and β–actin served as an internal control in all groups. The western blot images were quantified by ImageJ software (n = 3). (A) Before transfection, N2A parent cells showed no HSVtk expression level. After transfection and selection, the HSVtk expression levels of N2A-pCMV-HSVtk were stable within 4 weeks. Thus, N2A-pCMV-HSVtk was a stable clone and could be used for further experiments. (B) The U251 parent cells showed no HSVtk expression before transfection. U251-pCMV-HSVtk showed stable expression levels of HSVtk within 4 weeks and could be used as a control stable clone for glioblastoma. (C) HSVtk expression could not be detected in N2A-pCMV-HSVtk, since GFAP promoter was not activated in N2A cell line. (D) U251-pGFAP-HSVtk expressed HSVtk stably and continuously within 4 weeks.
(TIF)

S3 Fig Detection of GCV induced apoptosis of U251-pGFAP-HSVtk by TUNEL assay. (A-D) Fluorescence confocal images of apoptosis obtained from TUNEL assay. U251-pGFAP-HSVtk cells were treated with 5 μg/ml of GCV for 3 days. Both TUNEL- and Hoechst- positive cells were identified (C) and dying cells had rounded morphology in DIC image (D). (E-H) Positive control group was treated by 10 μM H_2_O_2_ for 1 hour before staining and the majority of the cell nuclei were TUNEL/Hoechst double positive. (I-L) U251-pGFAP-HSVtk cells without GCV treatment were regarded as negative control group and cell nuclei were TUNEL negative. Scale bar = 50 μm.
(TIF)

S4 Fig Cell viability assay by PI/Hoechst 33342 double nuclear staining for U251-pGFAP-HSVtk and U251-pCMV-HSVtk cells treated with GCV for 7 days. The survivals of U251-pGFAP-HSVtk (A-F) and U251-pCMV-HSVtk (G-L) cells were determined by PI/Hoechst co-staining for cell nuclei after 5 μg/ml GCV treatment for 7 days. PI/Hoechst co-localized and condensed cell nuclei were identified at the 5th day and the 7th day of GCV treatment in both U251-pGFAP-HSVtk (D and E, arrows) and U251-pCMV-HSVtk (J and K, arrows) groups. No PI signal was detected after 3 days of recovery (F and L). Scale bar = 50 μm.
(TIF)

S1 Table List of antibodies applied in this research. (DOCX)

S1 Raw images (PDF)

## References

[1] Gallego O. Nonsurgical treatment of recurrent glioblastoma. Current oncology (Toronto, Ont). 2015;22(4):e273–81. doi: 10.3747/co.22.2436 26300678 PMC4530825

[2] Bleeker FE, Molenaar RJ, Leenstra S. Recent advances in the molecular understanding of glioblastoma. Journal of neuro-oncology. 2012;108(1):11–27. doi: 10.1007/s11060-011-0793-0 22270850 PMC3337398

[3] Clarke MF, Fuller M. Stem cells and cancer: two faces of eve. Cell. 2006;124(6):1111–5. doi: 10.1016/j.cell.2006.03.011 16564000

[4] Tan BT, Park CY, Ailles LE, Weissman IL. The cancer stem cell hypothesis: a work in progress. Laboratory investigation; a journal of technical methods and pathology. 2006;86(12):1203–7. doi: 10.1038/labinvest.3700488 17075578

[5] Murat A, Migliavacca E, Gorlia T, Lambiv WL, Shay T, Hamou MF, et al. Stem cell-related "self-renewal" signature and high epidermal growth factor receptor expression associated with resistance to concomitant chemoradiotherapy in glioblastoma. Journal of clinical oncology: official journal of the American Society of Clinical Oncology. 2008;26(18):3015–24. doi: 10.1200/JCO.2007.15.7164 18565887

[6] Dalerba P, Cho RW, Clarke MF. Cancer stem cells: models and concepts. Annual review of medicine. 2007;58:267–84. doi: 10.1146/annurev.med.58.062105.204854 17002552

[7] Kondo T. Brain cancer stem-like cells. European journal of cancer (Oxford, England: 1990). 2006;42(9):1237–42. doi: 10.1016/j.ejca.2006.01.038 16632342

[8] Gilbertson RJ, Gutmann DH. Tumorigenesis in the brain: location, location, location. Cancer research. 2007;67(12):5579–82. doi: 10.1158/0008-5472.CAN-07-0760 17575119

[9] Reya T, Morrison SJ, Clarke MF, Weissman IL. Stem cells, cancer, and cancer stem cells. Nature. 2001;414(6859):105–11. doi: 10.1038/35102167 11689955

[10] Dragu DL, Necula LG, Bleotu C, Diaconu CC, Chivu-Economescu M. Therapies targeting cancer stem cells: Current trends and future challenges. World journal of stem cells. 2015;7(9):1185–201. doi: 10.4252/wjsc.v7.i9.1185 26516409 PMC4620424

[11] Linskey ME, Gilbert MR. Glial differentiation: a review with implications for new directions in neuro-oncology. Neurosurgery. 1995;36(1):1–21; discussion -2. doi: 10.1227/00006123-199501000-00001 7708144

[12] Zhuang W, Li B, Long L, Chen L, Huang Q, Liang Z. Induction of autophagy promotes differentiation of glioma-initiating cells and their radiosensitivity. International journal of cancer. 2011;129(11):2720–31. doi: 10.1002/ijc.25975 21384342

[13] Zhuang W, Long L, Zheng B, Ji W, Yang N, Zhang Q, et al. Curcumin promotes differentiation of glioma-initiating cells by inducing autophagy. Cancer science. 2012;103(4):684–90. doi: 10.1111/j.1349-7006.2011.02198.x 22192169 PMC7659256

[14] Long SSP, Larry K.; Prober, Charles G. Principles and Practice of Pediatric Infectious Disease: Elsevier Health Sciences; 2012.

[15] Matthews T, Boehme R. Antiviral activity and mechanism of action of ganciclovir. Reviews of infectious diseases. 1988;10 Suppl 3:S490–4. doi: 10.1093/clinids/10.supplement_3.s490 2847285

[16] Zhang JH, Wan MX, Pan BR, Yu B. Cytotoxicity of HSVtk and hrTNF-alpha fusion genes with IRES in treatment of gastric cancer. Cancer biology & therapy. 2004;3(11):1075–80. doi: 10.4161/cbt.3.11.1174 15477756

[17] Beck C, Cayeux S, Lupton SD, Dorken B, Blankenstein T. The thymidine kinase/ganciclovir-mediated "suicide" effect is variable in different tumor cells. Human gene therapy. 1995;6(12):1525–30. doi: 10.1089/hum.1995.6.12-1525 8664377

[18] Chen D, Tang Q. An experimental study on cervix cancer with combination of HSV-TK/GCV suicide gene therapy system and 60Co radiotherapy. BMC cancer. 2010;10:609. doi: 10.1186/1471-2407-10-609 21054886 PMC2988757

[19] Ahn YH, Yi H, Shin JY, Lee KD, Shin SP, Lee SJ, et al. STAT3 silencing enhances the efficacy of the HSV.tk suicide gene in gastrointestinal cancer therapy. Clinical & experimental metastasis. 2012;29(4):359–69. doi: 10.1007/s10585-012-9458-4 22350508

[20] Tamura R, Miyoshi H, Morimoto Y, Oishi Y, Sampetrean O, Iwasawa C, et al. Gene Therapy Using Neural Stem/Progenitor Cells Derived from Human Induced Pluripotent Stem Cells: Visualization of Migration and Bystander Killing Effect. Human gene therapy. 2020;31(5–6):352–66. doi: 10.1089/hum.2019.326 32075424

[21] Schuldiner M, Itskovitz-Eldor J, Benvenisty N. Selective ablation of human embryonic stem cells expressing a "suicide" gene. Stem cells (Dayton, Ohio). 2003;21(3):257–65. doi: 10.1634/stemcells.21-3-257 12743320

[22] Naujok O, Kaldrack J, Taivankhuu T, Jorns A, Lenzen S. Selective removal of undifferentiated embryonic stem cells from differentiation cultures through HSV1 thymidine kinase and ganciclovir treatment. Stem cell reviews and reports. 2010;6(3):450–61. doi: 10.1007/s12015-010-9148-z 20411442

[23] Inagaki M, Nakamura Y, Takeda M, Nishimura T, Inagaki N. Glial fibrillary acidic protein: dynamic property and regulation by phosphorylation. Brain pathology (Zurich, Switzerland). 1994;4(3):239–43. 10.1111/j.1750-3639.1994.tb00839.x 7952265

[24] Rutka JT, Murakami M, Dirks PB, Hubbard SL, Becker LE, Fukuyama K, et al. Role of glial filaments in cells and tumors of glial origin: a review. Journal of neurosurgery. 1997;87(3):420–30. doi: 10.3171/jns.1997.87.3.0420 9285609

[25] Tardy M, Fages C, Riol H, LePrince G, Rataboul P, Charriere-Bertrand C, et al. Developmental expression of the glial fibrillary acidic protein mRNA in the central nervous system and in cultured astrocytes. Journal of neurochemistry. 1989;52(1):162–7. doi: 10.1111/j.1471-4159.1989.tb10911.x 2908887

[26] Besnard F, Brenner M, Nakatani Y, Chao R, Purohit HJ, Freese E. Multiple interacting sites regulate astrocyte-specific transcription of the human gene for glial fibrillary acidic protein. The Journal of biological chemistry. 1991;266(28):18877–83. 1918004

[27] Brenner M, Kisseberth WC, Su Y, Besnard F, Messing A. GFAP promoter directs astrocyte-specific expression in transgenic mice. The Journal of neuroscience: the official journal of the Society for Neuroscience. 1994;14(3 Pt 1):1030–7. doi: 10.1523/JNEUROSCI.14-03-01030.1994 8120611 PMC6577554

[28] Vandier D, Rixe O, Brenner M, Gouyette A, Besnard F. Selective killing of glioma cell lines using an astrocyte-specific expression of the herpes simplex virus-thymidine kinase gene. Cancer research. 1998;58(20):4577–80. 9788604

[29] Li YF, Yuan YY, Zhang YM, Zhao N, Zhang Q, Meng FX, et al. HSVtk/GCV system on hepatoma carcinoma cells: Construction of the plasmid pcDNA3.1pAFP-TK and targeted killing effect. Molecular medicine reports. 2017;16(1):764–72. doi: 10.3892/mmr.2017.6657 28560395 PMC5482189

[30] Miura M, Tamura T, Mikoshiba K. Cell-specific expression of the mouse glial fibrillary acidic protein gene: identification of the cis- and trans-acting promoter elements for astrocyte-specific expression. Journal of neurochemistry. 1990;55(4):1180–8. doi: 10.1111/j.1471-4159.1990.tb03123.x 2398353

[31] Dekker J. Gene regulation in the third dimension. Science (New York, NY). 2008;319(5871):1793–4. doi: 10.1126/science.1152850 18369139 PMC2666883

[32] Sanyal A, Lajoie BR, Jain G, Dekker J. The long-range interaction landscape of gene promoters. Nature. 2012;489(7414):109–13. doi: 10.1038/nature11279 22955621 PMC3555147

[33] Roth DB, Wilson JH. Relative rates of homologous and nonhomologous recombination in transfected DNA. Proceedings of the National Academy of Sciences of the United States of America. 1985;82(10):3355–9. doi: 10.1073/pnas.82.10.3355 2987922 PMC397774

[34] Shcherbakova OG, Filatov MV. Camptothecin enhances random integration of transfected DNA into the genome of mammalian cells. Biochimica et biophysica acta. 2000;1495(1):1–3. doi: 10.1016/s0167-4889(99)00151-2 10634926

[35] Pasleau F, Tocci MJ, Leung F, Kopchick JJ. Growth hormone gene expression in eukaryotic cells directed by the Rous sarcoma virus long terminal repeat or cytomegalovirus immediate-early promoter. Gene. 1985;38(1–3):227–32. doi: 10.1016/0378-1119(85)90221-5 2998944

[36] Bak XY, Yang J, Wang S. Baculovirus-transduced bone marrow mesenchymal stem cells for systemic cancer therapy. Cancer gene therapy. 2010;17(10):721–9. doi: 10.1038/cgt.2010.32 20539321

[37] Pranjol MZ, Hajitou A. Bacteriophage-derived vectors for targeted cancer gene therapy. Viruses. 2015;7(1):268–84. doi: 10.3390/v7010268 25606974 PMC4306838

[38] Gotoh A, Ko SC, Shirakawa T, Cheon J, Kao C, Miyamoto T, et al. Development of prostate-specific antigen promoter-based gene therapy for androgen-independent human prostate cancer. The Journal of urology. 1998;160(1):220–9. 9628654

[39] Xiong J, Sun WJ, Wang WF, Liao ZK, Zhou FX, Kong HY, et al. Novel, chimeric, cancer-specific, and radiation-inducible gene promoters for suicide gene therapy of cancer. Cancer. 2012;118(2):536–48. doi: 10.1002/cncr.26289 21717442

[40] Azatian A, Yu H, Dai W, Schneiders FI, Botelho NK, Lord RV. Effectiveness of HSV-tk suicide gene therapy driven by the Grp78 stress-inducible promoter in esophagogastric junction and gastric adenocarcinomas. Journal of gastrointestinal surgery: official journal of the Society for Surgery of the Alimentary Tract. 2009;13(6):1044–51. doi: 10.1007/s11605-009-0839-1 19277794

[41] Kang TW, Choi SW, Yang SR, Shin TH, Kim HS, Yu KR, et al. Growth arrest and forced differentiation of human primary glioblastoma multiforme by a novel small molecule. Scientific reports. 2014;4:5546. doi: 10.1038/srep05546 24989033 PMC4080225

[42] Law BK. Rapamycin: an anti-cancer immunosuppressant? Critical reviews in oncology/hematology. 2005;56(1):47–60. doi: 10.1016/j.critrevonc.2004.09.009 16039868

[43] Cao X, Gu Y, Jiang L, Wang Y, Liu F, Xu Y, et al. A new approach to screening cancer stem cells from the U251 human glioma cell line based on cell growth state. Oncology reports. 2013;29(3):1013–8. doi: 10.3892/or.2012.2206 23258424

[44] Zhou ZH, Ping YF, Yu SC, Yi L, Yao XH, Chen JH, et al. A novel approach to the identification and enrichment of cancer stem cells from a cultured human glioma cell line. Cancer letters. 2009;281(1):92–9. doi: 10.1016/j.canlet.2009.02.033 19324493

[45] Hu W, Liu W. Side populations of glioblastoma cells are less sensitive to HSV-TK/GCV suicide gene therapy system than the non-side population. In vitro cellular & developmental biology Animal. 2010;46(6):497–501. 20135358 10.1007/s11626-010-9274-6

[46] Hu S, Zhao MT, Jahanbani F, Shao NY, Lee WH, Chen H, et al. Effects of cellular origin on differentiation of human induced pluripotent stem cell-derived endothelial cells. JCI insight. 2016;1(8). doi: 10.1172/jci.insight.85558 27398408 PMC4937999

[47] Mesnil M, Yamasaki H. Bystander effect in herpes simplex virus-thymidine kinase/ganciclovir cancer gene therapy: role of gap-junctional intercellular communication. Cancer research. 2000;60(15):3989–99. 10945596

[48] Roth JA, Cristiano RJ. Gene therapy for cancer: what have we done and where are we going? Journal of the National Cancer Institute. 1997;89(1):21–39. doi: 10.1093/jnci/89.1.21 8978404

[49] Asklund T, Appelskog IB, Ammerpohl O, Langmoen IA, Dilber MS, Aints A, et al. Gap junction-mediated bystander effect in primary cultures of human malignant gliomas with recombinant expression of the HSVtk gene. Experimental cell research. 2003;284(2):185–95. doi: 10.1016/s0014-4827(02)00052-6 12651152

[50] Gentry BG, Im M, Boucher PD, Ruch RJ, Shewach DS. GCV phosphates are transferred between HeLa cells despite lack of bystander cytotoxicity. Gene therapy. 2005;12(13):1033–41. doi: 10.1038/sj.gt.3302487 15789060

[51] Rubsam LZ, Boucher PD, Murphy PJ, KuKuruga M, Shewach DS. Cytotoxicity and accumulation of ganciclovir triphosphate in bystander cells cocultured with herpes simplex virus type 1 thymidine kinase-expressing human glioblastoma cells. Cancer research. 1999;59(3):669–75. 9973216

[52] Peltier J, O’Neill A, Schaffer DV. PI3K/Akt and CREB regulate adult neural hippocampal progenitor proliferation and differentiation. Developmental neurobiology. 2007;67(10):1348–61. doi: 10.1002/dneu.20506 17638387

