# Supplementary material for: Neural differentiation of glioblastoma cell lines via a herpes simplex virus thymidine kinase/ganciclovir system driven by a glial fibrillary acidic protein promoter
Source: PLoS One. 2021 Aug 9;16(8):e0253008. doi: 10.1371/journal.pone.0253008 (PMC8351974; doi:10.1371/journal.pone.0253008)
Supplement: S1 Table — (DOCX) [file pone.0253008.s005.docx]

## S1 Table. List of antibodies applied in this research.

| Antigen | Species | Working Dilution | Source | Catalog/  Clone No. |
| --- | --- | --- | --- | --- |
| **Primary Antibodies** |  |  |  |  |
| HSVtk | Goat | 1:200 (WB) | Santa Cruz | vL-20 |
| β-actin | Rabbit | 1:3000 (WB) | Abcam | ab8227 |
| Green fluorescence protein (GFP) | Mouse | 1:200 (ICC) | Millipore | MAB3580 |
| GFP | Rabbit | 1:200 (ICC) | Millipore | ab3080 |
| βIII-tubulin | Mouse | 1:400 (ICC) | Millipore | MAB5564 |
| Glial fibrillary acidic protein (GFAP) | Mouse | 1:300 (ICC) | Abcam | ab10062 |
| Nestin | Mouse | 1:200 (ICC) | Abcam | ab6142 |
| **Secondary Antibodies** |  |  |  |  |
| Anti-Rabbit IgG  (HRP-conjugated) | Goat | 1:5000 (WB) | Bethyl [Laboratories](http://www.bethyl.com/) | A120-101P |
| Anti-Goat IgG  (HRP-conjugated) | Donkey | 1:5000 (WB) | Bethyl [Laboratories](http://www.bethyl.com/) | A50-101P |
| Anti-Rabbit-IgG  (FITC-conjugated) | Goat | 1:200 (ICC) | Sigma-Aldrich | F9887 |
| Anti-Mouse-IgG  (FITC-conjugated) | Goat | 1:200 (ICC) | Sigma-Aldrich | F9006 |
| Anti-Rabbit-IgG  (TRITC-conjugated) | Goat | 1:200 (ICC) | Sigma-Aldrich | T5268 |
| Anti-mouse-IgG  (TRITC-conjugated) | Goat | 1:200 (ICC) | Sigma-Aldrich | T5393 |
