# Supplementary figures and images for: Neural differentiation of glioblastoma cell lines via a herpes simplex virus thymidine kinase/ganciclovir system driven by a glial fibrillary acidic protein promoter
Source: PLoS One. 2021 Aug 9;16(8):e0253008. doi: 10.1371/journal.pone.0253008 (PMC8351974; doi:10.1371/journal.pone.0253008)

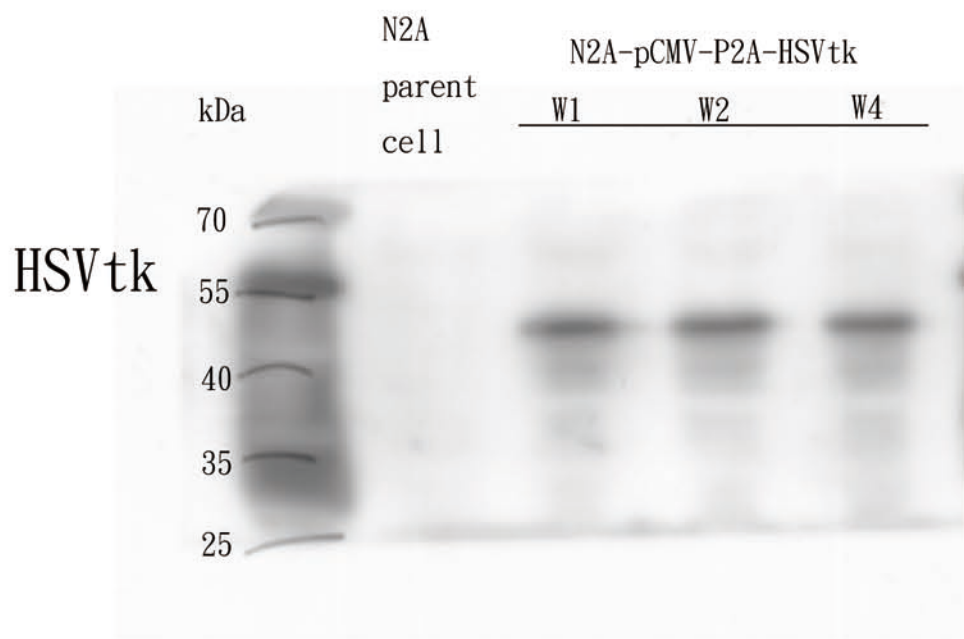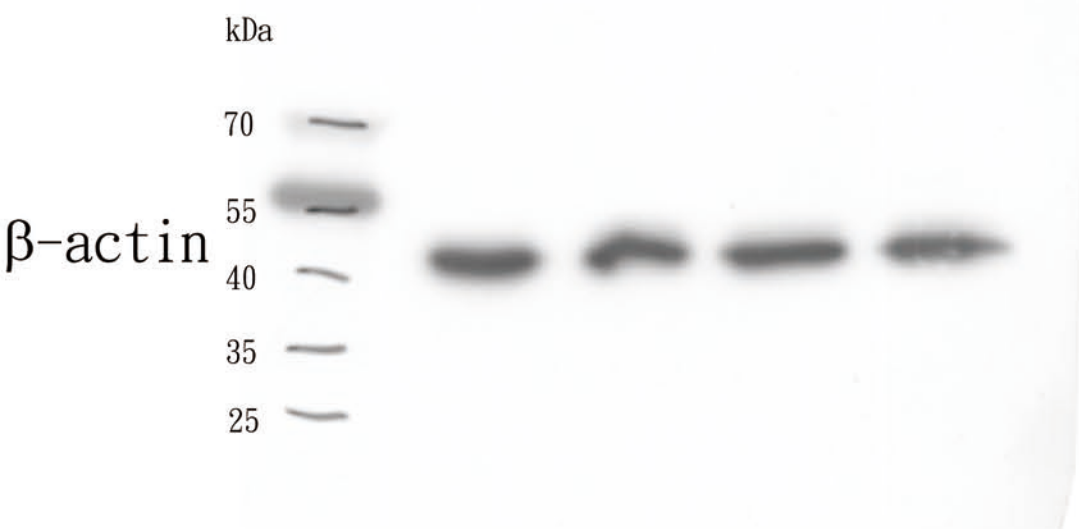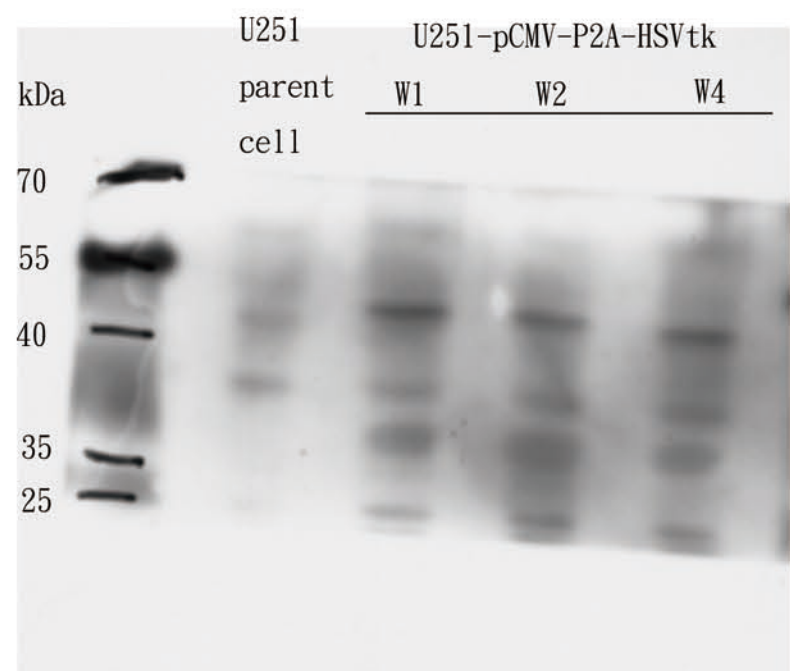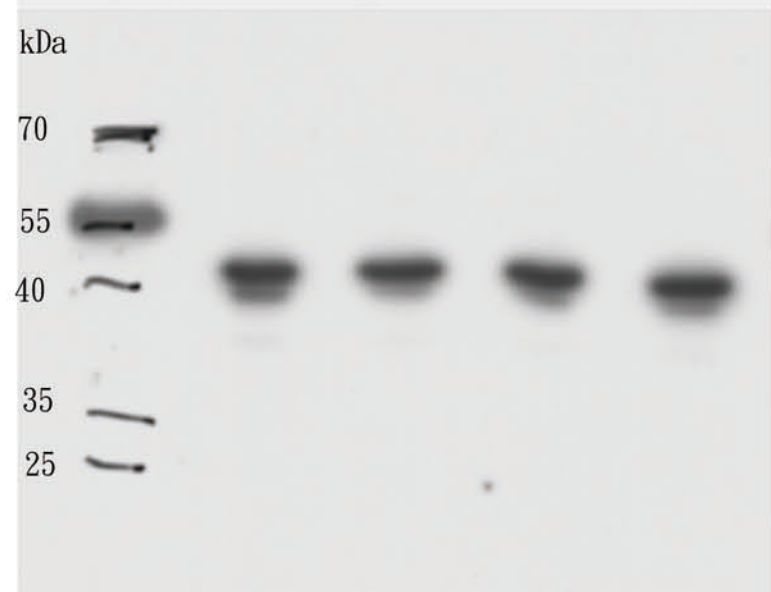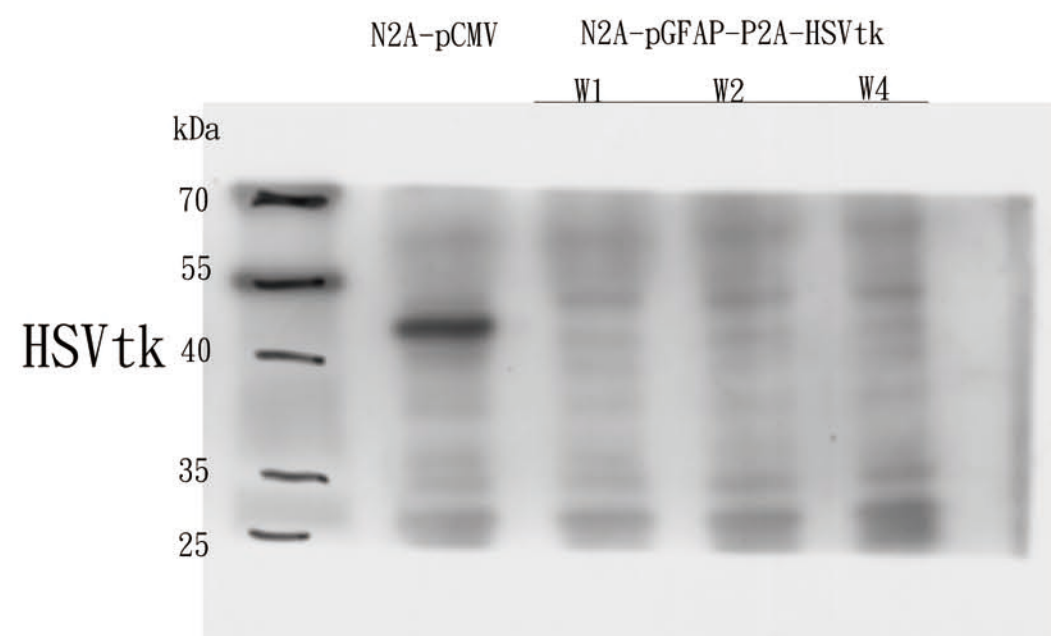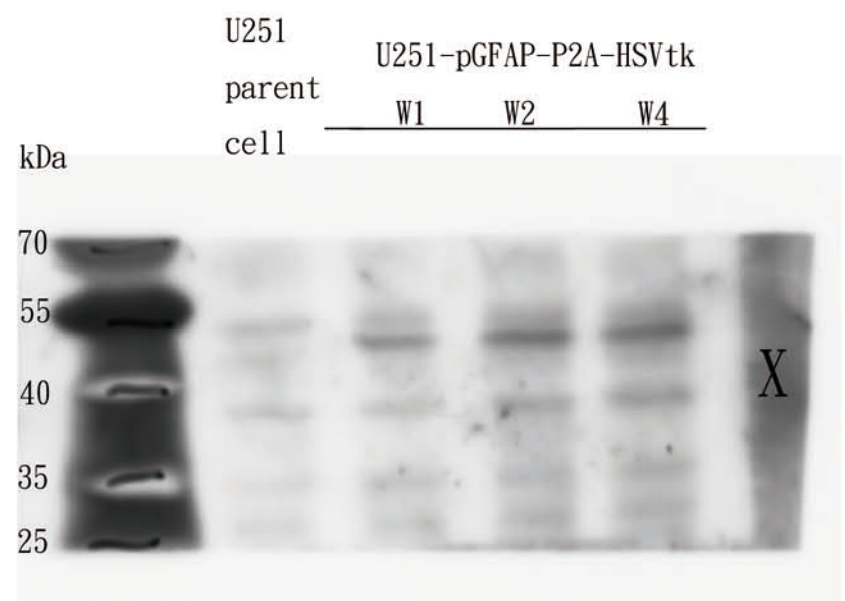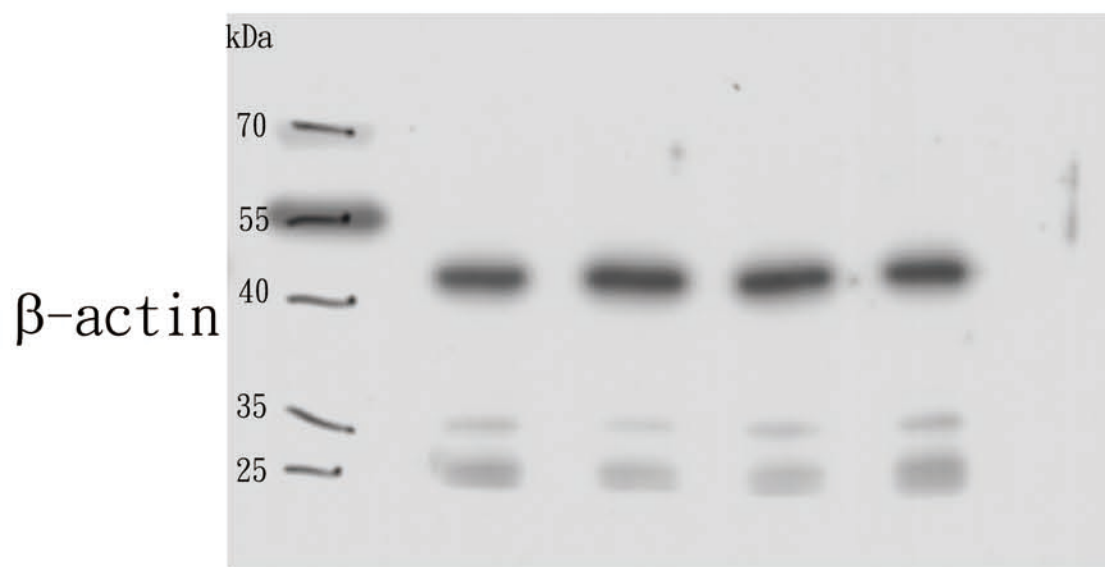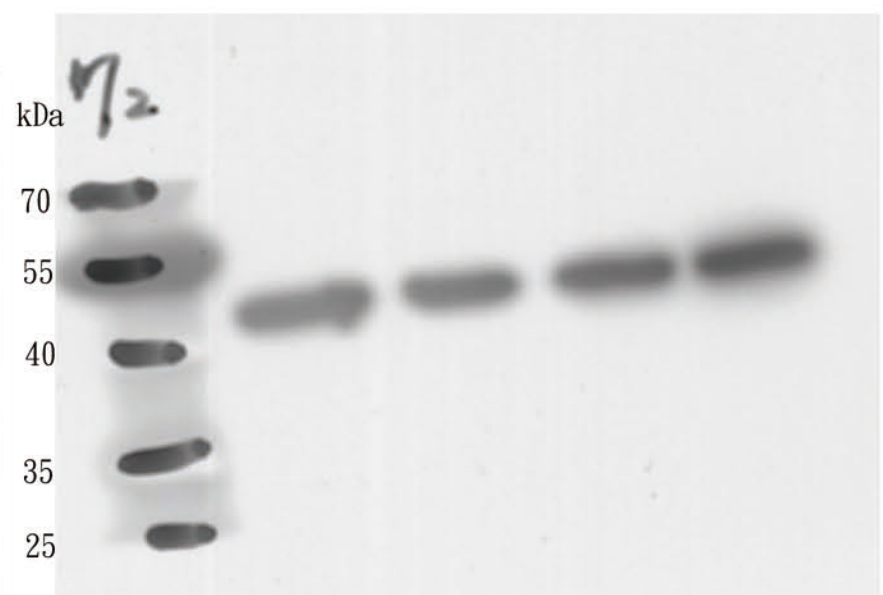

Supplement: S1 Raw images — (PDF) [file pone.0253008.s006.pdf]
